# Anti-Myocardial Ischemia Reperfusion Injury Mechanism of Dried Ginger-Aconite Decoction Based on Network Pharmacology

**DOI:** 10.3389/fphar.2021.609702

**Published:** 2021-05-06

**Authors:** Feng Xie, Yuan-Yuan Wu, Guang-Jing Duan, Bin Wang, Feng Gao, Pei-Feng Wei, Lin Chen, A-Ping Liu, Min Li

**Affiliations:** School of Pharmacy, Shaanxi University of Chinese Medicine, Xi’an, China

**Keywords:** dried ginger-aconite decoction, myocardial ischemia reperfusion injury, network pharmacology, energy metabolism, tcm

## Abstract

Dried ginger-aconite decoction (DAD) is a traditional Chinese medicine (TCM) formula that has been extensively used in the treatment of myocardial ischemia reperfusion injury (MI/RI). However, its specific mechanism against MI/RI has not been reported yet. Therefore, this paper studies the potential active components and mechanism of DAD against MI/RI based on network pharmacology and experimental verification. Sixteen active components of DAD were screened according to oral bioavailability and drug similarity indices. Through Cytoscape 3.7.0, a component-target network diagram was drawn, and potential active components of DAD against MI/RI were determined. Protein-protein interaction (PPI) and compound-target-pathway (C-T-P) networks were established through the software to discover the biological processes, core targets and core pathways of DAD against MI/RI. High Performance Liquid Chromatography (HPLC) analysis identified the presence of potentially active core components for network pharmacological prediction in DAD. It was found that DAD might have played a therapeutic role in anti-MI/RI by activating the PI3K/Akt/GSK-3β signaling pathway in order to reduce mitochondrial hypoxia injury and myocardial cell apoptosis. The network pharmacological prediction was validated by Hypoxia/reoxygenation(H/R) model *in vitro* and ligation model of the ligation of the left anterior descending branch *in vivo*. It was verified that DAD had activated PI3K/AKT/GSK-3β to reduce myocardial apoptosis and play a therapeutic function in MI/RI.

## Introduction

Myocardial ischemia-reperfusion injury (MI/RI) denotes the further destruction of the cardiac structure, and the further aggravation of metabolic dysfunction or even irreversible damage of the myocardial cell, following the restoration of blood supply of ischemic and anoxic myocardial tissue, which mainly involves re-expansion of myocardial infarction area and life-threatening arrhythmia (Lee et al., 2002; Raedschelders et al., 2012; Inoue, 2016). A common clinical cardiovascular disease, it has since developed into a killer ailment with high morbidity and mortality (Hausenloy and Yellon, 2013; Heusch, 2017). It typically occurs among the middle-aged and elderly population; however, as social competition becomes increasingly fierce, pressure on the youth has also increased, as they are also prone to develop myocardial ischemic diseases (Ingram et al., 2013; Han et al., 2018). Some studies indicate that 18 million people die of cardiovascular diseases every year globally, of which MI/RI incidence accounts for around 50% (Wei, 2017). MI/RI pathogenesis involves the interaction of multiple mechanisms, including vasoconstrictor release, non-reperfusion, deep inflammatory response, apoptosis and necrosis (Chen et al., 2020; Li et al., 2020a; Samiotis et al., 2021). Albeit not quite effective, the current treatment methods for MI/RI are percutaneous coronary intervention and the use of related thrombolytic drugs; nevertheless, MI/RI still has a high mortality rate worldwide. Therefore, research and attention on the mechanism of MI/RI have a considerable significance for its prevention and treatment.

**GRAPHICAL ABSTRACT:**
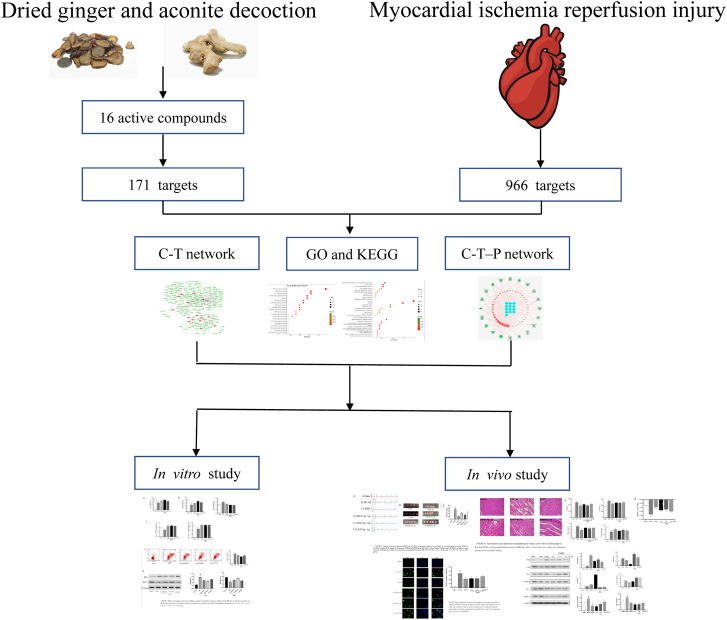
The graphical abstract of this study.

Traditional Chinese medicine (TCM) plays an indispensable role in the prevention and treatment of MI/RI. It spans a long history, including Yi Qi Huoxue decoction, Gualou Xiebai Baijiu decoction and Si Ni decoction (Deng et al., 2017; Zheng and Bao, 2017; Gao et al., 2019). It has been extensively used in MI/RI treatment, and dried ginger-aconite decoction (DAD), which comprises two kinds of Chinese herbal medicines, is one such medicine. Composed of aconite and dried ginger, DAD is recorded in the *Treatize on Febrile and Miscellaneous Disease.* Considering the efficacy of Yang for resuscitation, DAD is used to clinically treat ischemic heart diseases (Xu, 1986). Previous studies have indicated that DAD has a protective effect on the hearts of rats with MI/RI, and such effect is closely associated with its antioxidant and apoptosis effect (Shi et al., 2014). However, its bioactive compounds and their pharmacological mechanisms are still relatively unclear.

Network pharmacology integrates biological systems and multi-directional pharmacological approaches, incorporates biological networks and drug action networks, transcends the constraints of single-target beliefs, and begins from multi-target research strategies in order to achieve a comprehensive network analysis of drug effects (Xu et al., 2014; Chen et al., 2017; Zhang et al., 2018a). It is a significant approach to study the mechanisms of the multi-components, multi-targets and multi-pathways of TCM (Hopkins, 2008; Li and Zhang, 2013). The varied components of DAD are complex. Previous studies have determined that DAD can treat MI/RI by reducing the apoptosis of cardiomyocytes; however, the exact mechanism remains vague. Therefore, a comprehensive method is applied in this study to illustrate the molecular mechanisms of DAD. Network pharmacology is used to predict the active components and mechanisms of DAD in MI/RI treatment. HPLC is applied to determine whether DAD contains certain components for network pharmacological prediction. Afterward, *in vivo* and *in vitro* experiments are conducted to validate its mechanism on network pharmacological prediction. A graphical abstract of this study is presented in Graphical Abstract.

## Materials and Methods

### Materials

*Aconitum abietetorum* W.T.Wang and L.Q.Li (No. 51078020190334YC) and *Zingiber officinale Roscoe* (No. 51078020191020YC) were obtained from Jiangyou City, Sichuan Province, China. The geographical location of Jiangyou is within 31°32′26′′−32°19′18′′ north and 104°31′35′′−105°17′30′′ east. Material authentication for TCM identification was carried out by Professor Gang Zhang of Shaanxi University of Chinese Medicine. The samples were deposited at the Herbal Medicine Museum of the same university.

Fetal bovine serum (FBS) was purchased from BI (United States). Phosphate buffer saline (PBS) and Dulbecco’s modified Eagle medium (DMEM) were procured from Gibco (United States). Penicillinstreptomycin mixture and Cell Counting Kit-8 (CCK-8) from Shanghai Biyuntian Co., Ltd. (Shanghai, China). Dimethyl sulfoxide (DMSO) and trypsin were also procured from Gibco (United States). The assay kits for malondialdehyde (MDA), superoxide dismutase (SOD)apoptosis, atpase, creatine kinase (CK), mitochondrial permeability transition pore (MPTP), lactate dehydrogenase (LDH) and glutathione peroxidase (GSH-PS) were all obtained from Boster Biological Technology Co., Ltd. (Wuhan, China). Cyt-C, *β*-action, GADPH, Casp9, PI3K, AKT, Bax, Bcl-2 and phosphorylated(*P*)-AKT, GSK3β were also purchased from Boster Biological Technology Co., Ltd. (Wuhan, China). 6-gingerol (202,003), aconitine (No. A0608), mesaconitine (A0196) and hypaconitine (A0609) were all purchased from Chengdu Munst Biotechnology Co., Ltd. Standard purity was set as more than 98%. Methanol and triethylamine were purchased from Shaanxi Weitong Chemical Co., Ltd.

### Dried ginger-aconite decoction Preparation

Aconite and dried ginger were mixed at a 1:1 ratio. They were soaked in water for 0.5 h, then were boiled twice for 1 h each time. The filtrates were collected via gauzes, combined and concentrated to 1 g/ml to obtain the extract. For this study, 100 g of aconite and 100 g of dried ginger were prepared. Both components were completely immersed in water for 0.5 h. Then, 1.6 L water was added, letting the mixture boil for 1 h twice. The extract was then collected, filtered with gauze, and concentrated to 200 ml. The supernatant was obtained after centrifugation of the solution at 3,000 r/min, sterilized with 0.22 μm aqueous microporous membrane, and sealed.

### Network Pharmacology

#### Screening of Dried ginger-aconite decoction Active Components and Collection of Targets

The chemical constituents of aconite and dried ginger were examined from the Traditional Chinese Medicine Integrated Database (TCMSP, https://tcmspw.com/tcmsp.php) and the Comparative Toxicogenomic Database (CTD, http://ctdbase.org/), with aconite and dried ginger as the keywords. Active components of DAD were screened via oral bioavailability (OB) and drug-like quality (DL) (Cao et al., 2018), with DL ≥ 0.18 and OB ≥ 30% as the thresholds.

The primary compounds of aconite and dried ginger are alkaloids and volatile oil, both of which are irreplaceable and have good pharmacological activity. Thus, the following nine compounds were supplemented: deoxyaconitine, aconitine, hypaconitine, mesaconitin, 6-gingerol, 8-gingerol, 10-gingerol, 6-shogaol, and zingerone. The targets of all active compounds were obtained and imported into the Universal Protein (UniProt) database (https://www.uniprot.org/) to standardize their names.

#### Predicting Targets of DAD Against MI/RI

With “myocardial ischemia-reperfusion injury” as the keywords, MI/RI targets in the disgenet database (https://www.disgenet.org/) limited to “*Homo sapiens*” were obtained. The interactions of the DAD and MI/RI targets were considered as the potential therapeutic targets. The protein-protein interaction (PPI) of the common targets was accomplished in the string database (https://string-db.org/); the parameter organism was set to *Homo sapiens*, while the other basic settings were set as default. Using the Cytoscape 3.7.0 software, compound-target (C-T) and PPI were constructed.

#### Pathway and Functional Enrichment Analysis

The database for Annotation, Visualization and Integrated Discovery (DAVID) v6.8 (www.david.ncifcrf.gov/) provides a comprehensive set of functional annotation tools for researchers to understand the biological meanings behind extensive lists of genes. It was employed to undertake pathway enrichment analyses using the Gene Ontology (GO) and the Kyoto Encyclopedia of Genes and Genomes (KEGG) databases. Pathway terms with *p* < 0.05 were deemed significant. Using the Cytoscape 3.7.0 software, compound-target-pathway (C-T-P) was constructed.

### *In vitro* Experiment

#### HPLC Method for Component Analysis

DAD was filtered through a 0.22 μm nylon membrane prior to HPLC analysis. An HPLC System (Thermo, United States) was used to separate the components of DAD. All components were separated by Waters Bridge C18 (4.6 mm × 150 mm, 5 μm) and a C18 guard. Flowrate was set at 1.0 mL, min^−1^. The column temperature was 30°C. The wavelength was set at 237 nm. The mobile phases were (A) methanol and (B) triethylamine aqueous solution, with gradient elution of 0–15 min (A: B = 30:70), 15–40 min (A:B = 65:35) and baseline (A:B = 30:70).

#### Grouping and Modeling

Rat myocardial cells (H9C2) were purchased from Wuhan Punosai Life Science and Technology Co., Ltd. (Wuhan, China). The cells were cultured in DMEM with 10% FBS, 100 U/ml penicillin and 100 μl/ml streptomycin. They were maintained inside a humidified incubator with 95% air and 5% CO_2_ at 37°C. They were subjected to experimental procedures when they reached an 80% confluence level of population. They were classified into five groups: control group, Hypoxia/reoxygenation(H/R) group, DAD low-dose group (0.125 mg/ml), DAD medium-dose group (0.25 mg/ml), and DAD high-dose group (0.5 mg/ml). For all experiments, the cells were rendered quiescent by serum starvation for 24 h before treatment. Following pretreatment with DAD at varied doses for 24 h, the cells for all groups–except for the control group and the H/R group–were incubated in DMEM and glucose-free DMEM, respectively and then placed inside a hypoxia chamber (Stem Cell Technologies, San Diego, CA, United States). The chamber was flushed with 95% (v/v) N_2_ and 5% (v/v) CO_2_ at a flowrate of 15 L/min for 10 min, and maintained at 37°C to induce hypoxia injury. After hypoxia for 12 h, reoxygenation was conducted by replacing the medium to DMEM that contained 4.5 mM glucose (pH 7.4) and by subsequent incubation in a CO_2_ incubator (5% (v/v) CO_2_, 95% (v/v) air) for 2 h (Wang et al., 2018).

#### Survival Rate of H9C2 Cells

CCK8 assay was applied to determine the influence of DAD on the survival rate of H9C2 cells that were damaged by oxygen. The cells were briefly seeded onto 96-well plates and then cultured until they adhered. Afterward, the cells were treated with DAD at varied concentrations (0.125 mg/ml, 0.25 mg/ml, 0.5 mg/ml). Model group and control group were given DMEM (without glucose) and DMEM, respectively. Model according to the above method. Afterward, 10 μl of CCK-8 was added, and the mixture was incubated for another 2 h. Absorbance was recorded at 450 nm, and the experiments were performed in parallel in triplicate.

### Detection of Apoptosis Rate

The H/R damaged cells in each group were digested with 0.25% trypsin and centrifuged at 1,500 r/min for 5 min. The supernatant was discarded and the cells were collected. The collected cells were then resuspended with PBS (pH 7.2), washed with PBS twice, and centrifuged at 1,500 r/min for 5 min, before the supernatant was discarded. The precipitated cells were resuspended with 500 μl of binding buffer, then 5 μl of annexin V-FITC and 5 μl of PI staining solution was added. After mixing, the cells were incubated at room temperature in the dark for 5–15 min. Finally, the apoptosis for each group was detected by flow cytometry (NovoCyte 452180529501, Thermo, United States).

#### Biochemical Testing

After H/R injury, the cells in each group were obtained. According to the manufacturer’s protocols, the SOD, MDA, Na^+^-K^+^-ATP and Ca^2+^-Mg^2+^-ATP levels were detected by their respective commercial kits.

### *In vivo* Experiment

#### Establishment and Grouping of MI/RI in Rat Models

Sixty Sprague-Dawley male rats were purchased from Chengdu Da Shuo Experimental Co., Ltd. (Sichuan, China). They were housed in a specific-pathogen-free (SPF) environment. The rats in DAD low-dose, DAD medium-dose and DAD high-dose groups were orally administered with 1.4 g/kg, 2.8 g/kg and 5.6 g/kg DAD once daily, respectively. Those in the positive control group were orally administered with 0.09 g/kg per day of compound danshen dripping pills (CDDP), and those in the model, and sham group were orally administered with the same volume of 0.9% NaCl. A week later, all rats were operated, with the sham group only opening the chest without ligation. Left thoracotomy and pericardiectomy, followed by left anterior descending coronary artery ligation, were performed. After 40 min of ischemia, the ligature was opened for reperfusion for 2 h. The serum and heart tissue samples were prepared for future experiment. All animal experiments were performed in accordance with the Animal Care and Use Committee of the Institute of Materia Medica, China (No. TCM-2019–194,040-E08).

#### Detection of Myocardial Infarction Area in MI/RI Rats

Prior to the experiment, 2% TTC was placed in a 37°C thermostat for 0.5 h. Four rats were randomly selected from each group. Their hearts were removed, flushed with PBS, and rapidly frozen at −20°C. The specimens were uniformly cut into 1 mm slices under the line of ligature and placed in a 37°C, 2% TTC solution to dye for 20°min, and then fixed with 10% formaldehyde solution. Ultimately, the myocardial infarction area was white and the non-infarction area was red. The infarct area was calculated using ImageJ software (Media Cybernetics, Inc., Rockville, MD, United States). The applied equation was as follows,

$$\text{Infarction Range}=\frac{\text{Infarction Range}}{\text{Left Ventricular Area}}\times 100\%.$$

#### Immunohistochemical Staining

Immediately after reperfusion, the heart was removed and rinsed in precooled saline. The myocardium from the anterior wall of the left ventricle was removed. The heart was then fixed with precooled 4% paraformaldehyde and rinsed with water for 12 h. The specimens were dehydrated afterward. They were then immersed in xylene, and hematoxylin-eosin (H-E) staining was conducted after routine paraffin-embedded staining. Then, the slices were sealed with conventional resin, and the pathological changes in the myocardium were observed under an optical microscope (Olympus BX 41, Japan).

#### Myocardial Tissue Apoptosis Detection

After MI/RI modeling, the heart tissue was removed. The tissue sections were washed in a phosphate buffer solution, and fixed in a 4% paraformaldehyde solution. They were then cut into paraffin sections with a thickness of 4 μm, and proteinase K was added. After a strict color rendering according to the kit instructions, five visual fields were randomly selected for shooting, and the color images of ten independent fields were randomly captured and digitized. The cells with clear nuclear markers were defined as TUNEL positive. Image J software was used for recording, and the apoptosis rate was calculated. The applied equation was as follows:

$$\text{Apoptosis index}=\frac{\text{Number of TUNEL positive cells}}{\text{Total number of cardiomyocytes}}\times 100\%.$$

#### Biochemical Testing

After reperfusion, the rats were intraperitoneally anesthetized using chloral hydrate (30 mg/kg), and the blood samples were obtained from the abdominal aorta. The samples were left standing at room temperature for 30 min and then centrifuged at 3,000 r/min for 15 min. The serum was collected and stored at −80°C until used. Based on the manufacturer’s protocols, the GSH-Sp, MDA, CK and LDH levels were detected by the respective commercial kits.

#### Detection of MPTP Open Holes in Myocardial Tissues

The fresh myocardial tissue just removed was rinsed with PBS; the excess water on the surface of the myocardial tissue was absorbed using a filter paper. The proper part of the entire heart tissue was taken; its mass was accurately measured, and the tissue homogenate was prepared using a mass-to-volume ratio of 1:9. The entire operation needed to be conducted in an ice bath. Finally, the tissue homogenate was centrifuged for 3,500 r/min for 10 min. The supernatant was collected and stored at −80°C for later use. The openness of the MPTP holes in the homogenate was determined according to the kit instructions.

#### Western Blot Analysis (*in vivo* and *in vitro*)

The myocardial tissue and the H9C2 cells were lyzed by RIPA buffer (Shanghai Weiao Biological Technology Co., Ltd., China) containing cocktail protease inhibitors (1:100) and a protein phosphatase inhibitor (1:50) for 30 min on ice. The protein concentration in the supernatants was determined by BCA assay (Shanghai Weiao Biological Technology Co., Ltd., China). Protein samples were loaded with 10% SDS-polyacrylamide gel (Shanghai Weiao Biological Technology Co., Ltd. China), and then electrophoretically transferred onto PVDF (Millipore. Billerica, MA, United States). The membranes were blotted with 5% fat-free milk in a TBST buffer for 2 h at room temperature and then incubated at 4°C overnight with the following primary antibodies: anti-Caspase-9 (1:600), anti-Bax (1:500), anti-Bcl-2 (1:500), anti-Cyt-c (1:500), anti-PI3K (1:500), anti-Akt (1:1,000), anti-*p*-Akt (1:1,000), anti-*p*-GSK-3β (1:1,000), and anti-GAPDH (1:1,000). The membrane was rinsed thrice on the second day and then incubated with HRP-conjugated secondary antibodies for 1 h at room temperature. The blots were imaged under an enhanced chemiluminescence (ECL) system. The target band molecular weights and the net optical density were analyzed using the AlphaEase FC software (Alpha Innotech, United States).

### Statistical Analysis

All data were expressed as mean ± standard deviation (SD). GraphPad Prism 7 software was employed to ascertain statistically significant differences. The differences among multiple groups were assessed using one-way analysis of variance (ANOVA). The difference between the means was considered statistically significant when *p* < 0.05.

## Results

### Network Pharmacology

#### DAD Active Compounds and Target Screening

**FIGURE 1:**
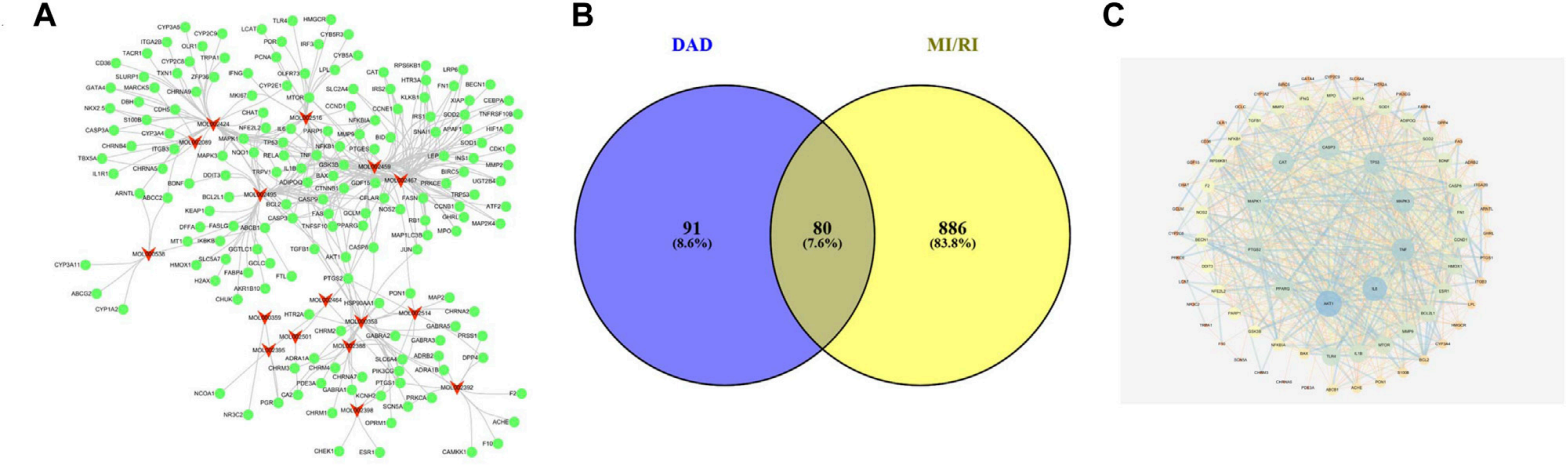
The networks of dried ginger and aconite decoction anti-MI/RI. **(A)** The compound-target network of DAD. The red nodes represent active compounds and the green nodes represent targets. The target surrounding the active components are proportional to their degree. **(B)** Overlap of DAD and MI/RI targets. The blue circles represent DAD targets and the yellow circles represent MI/RI targets. The shaded area is the target of DAD anti-MI/RI. **(C)** The protein-protein interaction network of protein targets obtained from STRING database and constructed by Cytoscape. The colors of the nodes are illustrated from blue to yellow to orange in descending order of degree values.

From aconite and dried ginger, 16 compounds (Table 1) were retrieved from the TCMSP database, and 171 targets were retrieved from the TCMSP and CTD databases (Figure 1A). A total of 966 targets of MI/RI were obtained from the DisGeNet databases. A total of 80 targets (Table 2) were obtained through the intersection of the 966 MI/RI targets and the 171 putative targets of aconite and dried ginger. These 80 mutual targets were identified as potential therapeutic targets for DAD against MI/RI (Figure 1B). The C-T network included 187 nodes (16 for potential bioactive components and 171 for protein targets). Among the bioactive components, aconitine (DAD, degree = 48), 6-ginger (DAD, degree = 31) and mesaconitine (DAD, degree = 25), hypaconitine (DAD, degree = 24) exhibited the greatest correlation with MI/RI. These could be the key components of DAD against MI/RI.

**TABLE 1:** Information on the 16 active compounds in the DAD.

Herbal name	TCMSP ID	Compound	OB	DL
Aconite	MOL002395	Deoxyandrographolide	56.3	0.31
Aconite	MOL002398	Karanjin	69.56	0.34
Aconite	MOL002424	aconitine	7.87	0.23
Aconite	MOL000538	hypaconitine	31.39	0.26
Aconite	MOL002089	mesaconitin	8.7	0.25
Aconite	MOL002388	Delphin_qt	57.76	0.28
Aconite	MOL002392	Deltoin	46.69	0.37
Dried ginger	MOL002467	6-gingerol	35.64	0.16
Dried ginger	MOL002459	10-gingerol	19.14	0.28
Dried ginger	MOL002495	6-shogaol	31	0.14
Dried ginger	MOL002516	zingerone	25.23	0.05
Dried ginger	MOL000359	sitosterol	36.91	0.75
Dried ginger	MOL002464	1-Monolinolein	37.18	0.3
Dried ginger	MOL002501	[(1S)-3-[(E)-but-2-enyl]-2-methyl-4-oxo-1-cyclopent-2-enyl] (1R,3R)-3-[(E)-3-methoxy-2-methyl-3-oxoprop-1-enyl]-2,2-dimethylcyclopropane-1-carboxylate	62.86	0.3
Dried ginger	MOL002514	Sexangularetin	35.64	0.16
Dried ginger	MOL000358	beta-sitosterol	36.91	0.75

**TABLE 2:** Targes information of DAD anti-MI/RI.

Target name	Full name of the target	Uniprot ID
MAPK3	Mitogen-activated protein kinase 3	P27361
CYP2C9	Cytochrome P450 2C9	P11712
CYP2C8	Cytochrome P450 2C8	P10632
CYP3A4	Cytochrome P450 3A4	P08684
ARNTL	Aryl hydrocarbon receptor nuclear translocator-like protein 1	O00327
CD36	Platelet glycoprotein 4	P16671
GATA4	Transcription factor GATA-4	P43694
ITGA2B	Integrin alpha-IIb	P08514
ITGB3	Integrin beta-3	P05106
MTOR	Serine/threonine-protein kinase mtor	P42345
OLR1	Ox-LDL receptor 1	P78380
S100B	Protein S100-B	P04271
TNF	Tumor necrosis factor	P01375
BAX	Apoptosis regulator BAX	Q07812
BCL2	Apoptosis regulator Bcl-2	P10415
BDNF	BDNF	P23560
CASP3	Caspase-3	P42574
MAPK1	Mitogen-activated protein kinase 1	P28482
CHAT	SH2 domain-containing protein 3C	Q8N5H7
CHRNA5	Neuronal acetylcholine receptor subunit alpha-5	P30532
IL1B	Interleukin-1 beta	P01584
IL6	Interleukin-6	P05231
NFKB1	Nuclear factor NF-kappa-B p105 subunit	P19838
TP53	Cellular tumor antigen p53	P04637
TRPA1	Transient receptor potential cation channel subfamily a member 1	O75762
ABCB1	ATP-dependent translocase ABCB1	P08183
CYP1A2	Cytochrome P450 1A2, EC 1.14.14.1	P05177
GSK3B	Glycogen synthase kinase-3 beta, GSK-3 beta	P49841
CCND1	G1/S-specific cyclin-D1	P24385
PPARG	PPAR-gamma	P37231
PTGS2	Prostaglandin G/H synthase 2	P35354
BIRC5	Baculoviral IAP repeat-containing protein 5	O15392
GDF15	Growth/differentiation factor 15	Q99988
CASP8	Caspase-8	Q14790
NOS2	Nitric oxide synthase, inducible	P35228
CAT	Catalase	P04040
MMP2	72 kDa type IV collagenases	P08253
ADIPOQ	Adiponectin	Q15848
MMP9	Matrix metalloproteinase-9	P14780
MPO	Myeloperoxidase	P05164
PARP1	Poly [ADP-ribose] polymerase 1	P09874
SOD1	Superoxide dismutase [Cu-Zn]	P00441
SOD2	Superoxide dismutase [Mn], mitochondrial	P04179
AKT1	RAC-alpha serine/threonine-protein kinase	P31749
BECN1	Beclin-1	Q14457
FAS	Tumor necrosis factor receptor superfamily member 6	P25445
FN1	Fibronectin	P02751
GHRL	Appetite-regulating hormone	Q9UBU3
HIF1A	Hypoxia-inducible factor 1-alpha	Q16665
NFKBIA	NF-kappa-B inhibitor alpha	P25963
PRKCE	Protein kinase C epsilon type	Q02156
RPS6KB1	Ribosomal protein S6 kinase beta-1	P23443
NFE2L2	Nuclear factor erythroid 2-related factor2	Q16236
HMOX1	Heme oxygenase 1	P09601
DDIT3	DNA damage-inducible transcript 3 protein	P35638
GCLC	Glutamate--cysteine ligase catalytic subunit	P48506
FABP4	Fatty acid-binding protein	P15090
GCLM	Glutamate--cysteine ligase regulatory subunit	P48507
BCL2L1	Bcl-2-like protein 1	Q07817
HMGCR	3-hydroxy-3-methylglutaryl-coenzyme a reductase	P00347
IFNG	Interferon gamma	P01579
LCAT	Phosphatidylcholine-sterol acyltransferase	P04180
LPL	Lipoprotein lipas	P06858
TGFB1	Transforming growth factor beta-1 proprotein	P01137
TLR4	Toll-like receptor 4	O00206
PTGS1	Prostaglandin G/H synthase 1	P23219
PIK3CG	PI3K-gamma	P48736
F2	Prothrombin	P00734
SCN5A	Sodium channel protein type 5 subunit alpha	Q14524
F10	Coagulation factor X	P00742
ACHE	Acetylcholinesterase, AChE, EC 3.1.1.7	P22303
ADRB2	Beta-2 adrenergic receptor	P07550
DPP4	Dipeptidyl peptidase 4	P27487
ESR1	Estrogen receptor	P03372
NR3C2	Mineralocorticoid receptor	P08235
CHRM3	Muscarinic acetylcholine receptor M3	P20309
PDE3A	cGMP-inhibited 3′,5′-cyclic phosphodiesterase A	Q14432
HTR2A	5-hydroxytryptamine receptor 2A	P28223
SLC6A4	Sodium-dependent serotonin transporter	P31645
PON1	Serum paraoxonase/arylesterase 1	P27169

#### PPI Network Analysis

To examine the potential interactions of the 80 targets, String 11.0 database was used to build a PPI network. The minimum combined score between the targets was set as the medium confidence (0.400). The PPI network of the potential target was saved as a TSV file and then entered into Cytoscape 3.7.0 for visualization (Figure 1C). In the PPI network, targets with high degrees played a significant role in central correlation. The top 5 targets, which were ranked in terms of degree value, were acquired as the core targets. These targets were AKT1 (degree = 47), IL6 (degree = 41), TNF (degree = 38), MAPK3 (degree = 36) and TP53 (degree = 30).

#### GO Enrichment Analysis

**FIGURE 2:**
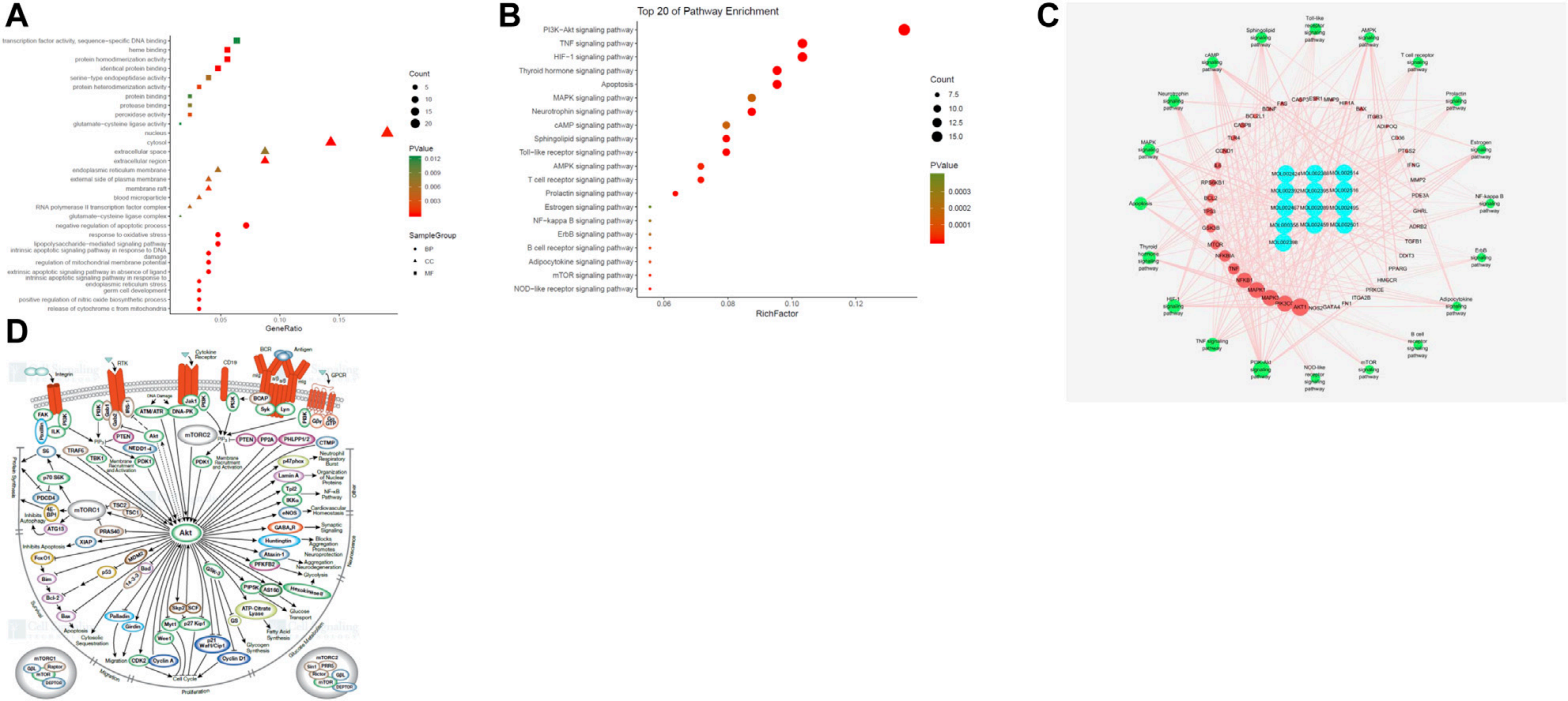
**(A)** The gene ontology (GO) enrichment analysis for key targets. **(B)** The KEGG pathway enrichment analysis of key targets. **(C)** The compound-target-pathway network constructed by Cytoscape. The blue nodes represent active components in DAD, the red nodes represent putative targets, the green nodes represent the signaling pathways. Node’s size is proportional to their degree. **(D)** PI3K/ Akt signaling pathway network (PI3K/Akt signaling pathway network is derived from https://www.cellsignal.cn/pathways/pathways-akt-signaling)

The biological function of DAD against MI/RI was identified by GO enrichment of the 80 potential therapeutic targets. A total of 158 GO items were obtained from the GO enrichment analysis of 80 potential therapeutic targets, including 118 biological processes (BP), 22 cell components (CC) and 18 molecular functions (MF) (*p* < 0.05). To realize a brief demonstration, only the top 10 significant GO entries were selected for further analysis. The top ten analyses for BP, CC and MF were selected respectively (Figure 2A), which indicated that DAD might regulate cell apoptosis, inflammation and mitochondrial energy metabolism to exert its therapeutic effects against MI/RI.

#### Pathway Enrichment

To examine the potential pathways of DAD on MI/RI, a pathway enrichment of the 80 potential therapeutic targets was conducted. The top 20 significantly enriched pathways are presented in Figure 2B. Among the potential pathways, PI3K/AKT signaling was the most prominently enriched based on the gene numbers. To further clarify and elucidate the molecular mechanism of DAD treatment on MI/RI, a C-T-P network diagram was drawn based on the top 20 signaling pathways, as well as the targets and compounds involved (Figure 2C). After integrating drug target predictions, pathway and function enrichments, and network analyses, AKT1, PIK3G, MAPK3, MAPK1, NFKB, TNF, NFKBA, MTOR, GSK3β and TP53 were identified. These targets were highly associated with apoptosis and inflammation. Likewise, they were considered as the key targets of DAD against MI/RI. Interestingly, of the aforementioned targets, only GSK3β was downstream of the PI3K/AKT signaling pathway (Figure 2D). Thus, it was speculated that the anti-MI/RI effect of DAD might be associated with its regulation of apoptosis and mitochondrial energy metabolism by targeting PI3K/AKT/GSK3β signaling pathways with their relevant activators.

### *In vitro* Experiments

#### HPLC Analysis

Network pharmacology predicted that aconitine, 6-ginger, mesaconitine and hypaconitine in DAD were the potential active components of anti-MI/RI in DAD. The phytochemical composition of DAD was assessed using HPLC. As shown in Supplementary Figure S1, DAD contained aconitine, 6-ginger, mesaconitine.

#### H9C2 Cells’ Survival Test Results

**FIGURE 3:**
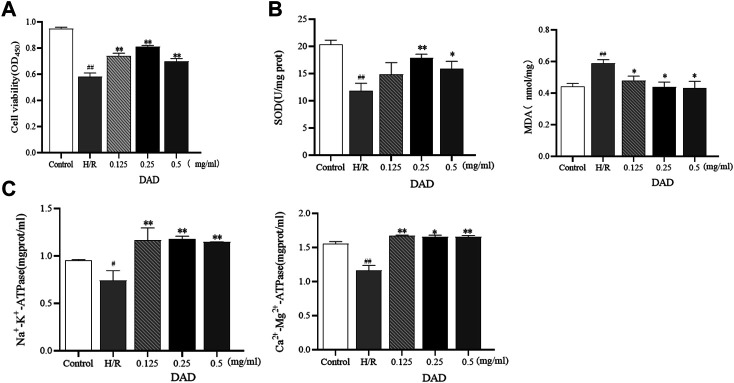
Effects of dried ginger-aconite decoction (DAD) on survival rate and biochemical parameters of H9C2 cells damaged by H/R. **(A)** Effect on the survival rate of H9C2 cells. **(B)** Effect on oxidative stress factors MDA, SOD. **(C)** The effect on the activity of ATPase. Data were presented as mean standard deviation (SD). #p < 0.05, ##p < 0.01 vs. control group. *p < 0.05, **p < 0.01 vs. H/R group.

The effects of DAD were initially assessed based on the cell viability of the H9C2 cells damaged by H/R. It was found that the exposure of H9C2 cells to H/R injury had led to a decrease in cells (*p* < 0.01). Compared to the control group, the survival rate of the H/R group was only 58%. When the H9C2 cells were pretreated with 0.125–0.5 mg/ml DAD, cell viability was significantly restored (*p* < 0.01). DAD (0.25 mg/ml) had the greatest effect on cell survival rate, which increased by 25% (*p* < 0.01), compared to the H/R group (Figure 3A). These data suggest that DAD pretreatment may provide protection against H/R-induced cardiomyocyte injury.

#### Results of Biochemical Testing

The outcome of cardiomyocyte hypoxia was insufficient oxygen as required by the mitochondria, which would lead to mitochondrial damage, reduced ATP production and aggravated oxidative damage of the cardiomyocytes. SOD is an oxygen free radical scavenger in human body (Khatua et al., 2012; Ling et al., 2019). The final product of oxidative damage is MDA, which can damage the mitochondria. The change in MDA can reflect the degree of oxidative damage of the cells (Mao et al., 2008; Radmanesh et al., 2017). The enzymatic activities of Na^+^-K^+^-ATP and Ca^2+^-Mg^2+^-ATP–indirectly reflect Changes in the amount of ATP (Zhu et al., 2019). After pretreatment with varied DAD doses, the MDA levels of H9C2 cells damaged by H/R could be reduced to varied degrees, as well as increased the activities of SOD, Na^+^-K^+^-ATP and Ca^2+^-Mg^2+^-ATP. In the administration group (Figure 3B-C), DAD (0.25 mg/mg) manifested the best therapeutic effect (*p* < 0.05). These results imply that the protective effect of DAD on H/R-damaged H9C2 is related to the mitochondria.

#### Effect of DAD on the Apoptosis Rate of H9C2 Cells With H/R Injury

**FIGURE 4:**
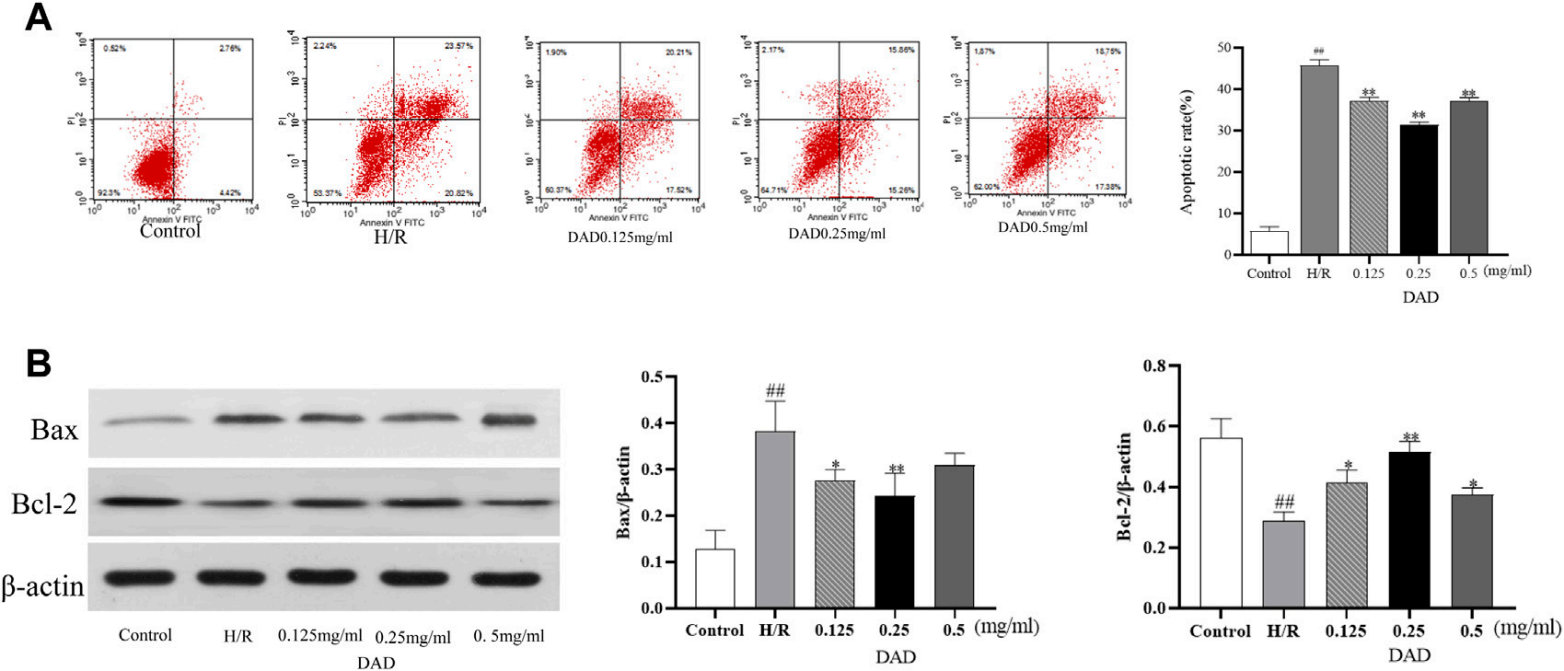
Effects of dried ginger-aconite decoction (DAD) on apoptosis rate and Bax/Bcl-2 expression of H9C2 cells after H/R injury. **(A)** The effect on apoptosis rate. **(B)** The effect on the expression of Bax/Bcl-2. Data were presented as mean standard deviation (SD) of three independent experiments. #p < 0.05, ##p < 0.01 vs. control group. *p < 0.05, **p < 0.01 vs. H/R group.

As discussed, ischemia and hypoxia aggravate the oxidative damage of cardiomyocytes and eventually induce the apoptosis of cardiomyocytes. H/R injury significantly increased the apoptosis rate of the H9C2 cells, which increased by 35% compared to the control group (*p* < 0.01). after DAD preconditioning. The apoptosis rate of the H9C2 cells damaged by H/R significantly decreased, while the apoptosis rate of the DAD group (0.25 mg/kg) decreased by 18% (*p* < 0.01) compared to the H/R group (Figure 4A).

#### Western Blot Analysis

Network pharmacological analysis implied that the molecular mechanism of the anti-MI/RI effect of DAD might be associated with apoptosis. The mammalian BCL-2 family member Bcl-2 was an anti-apoptotic protein, while Bax protein induced apoptosis by enhancing cytochrome c (Cyt-C) release from the mitochondria (Aamazadeh et al., 2020; Lin et al., 2020). Therefore, the two targets of Bax and Bcl2 (Bax and Bcl2 belonged to the targets of DAD in anti-MI/RI) were validated *in vitro*. Compared to the control group, the expression of Bax had significantly increased and the expression of Bcl-2 had significantly decreased after the H9C2 cells were damaged by H/R (*p* < 0.01). Compared to the H/R group, the expression of Bax in the H9C2 cells damaged by H/R had significantly decreased, while the expression of BCL2 had significantly increased when the H9C2 cells were pretreated by DAD (*p* < 0.01). In the administration group (Figure 4B), DAD (0.25 mg/mg) manifested the best therapeutic effect. *In vitro* studies were found consistent with network pharmacology, with DAD being shown to resist MI/RI by reducing myocardial cell apoptosis. *In vitro* studies were found consistent with network pharmacology, with DAD being shown to resist MI/RI by reducing myocardial cell apoptosis.

### *In vivo* Experiments

#### Results of ECG and Myocardial Infarction Area in MI/RI Rats

**FIGURE 5:**
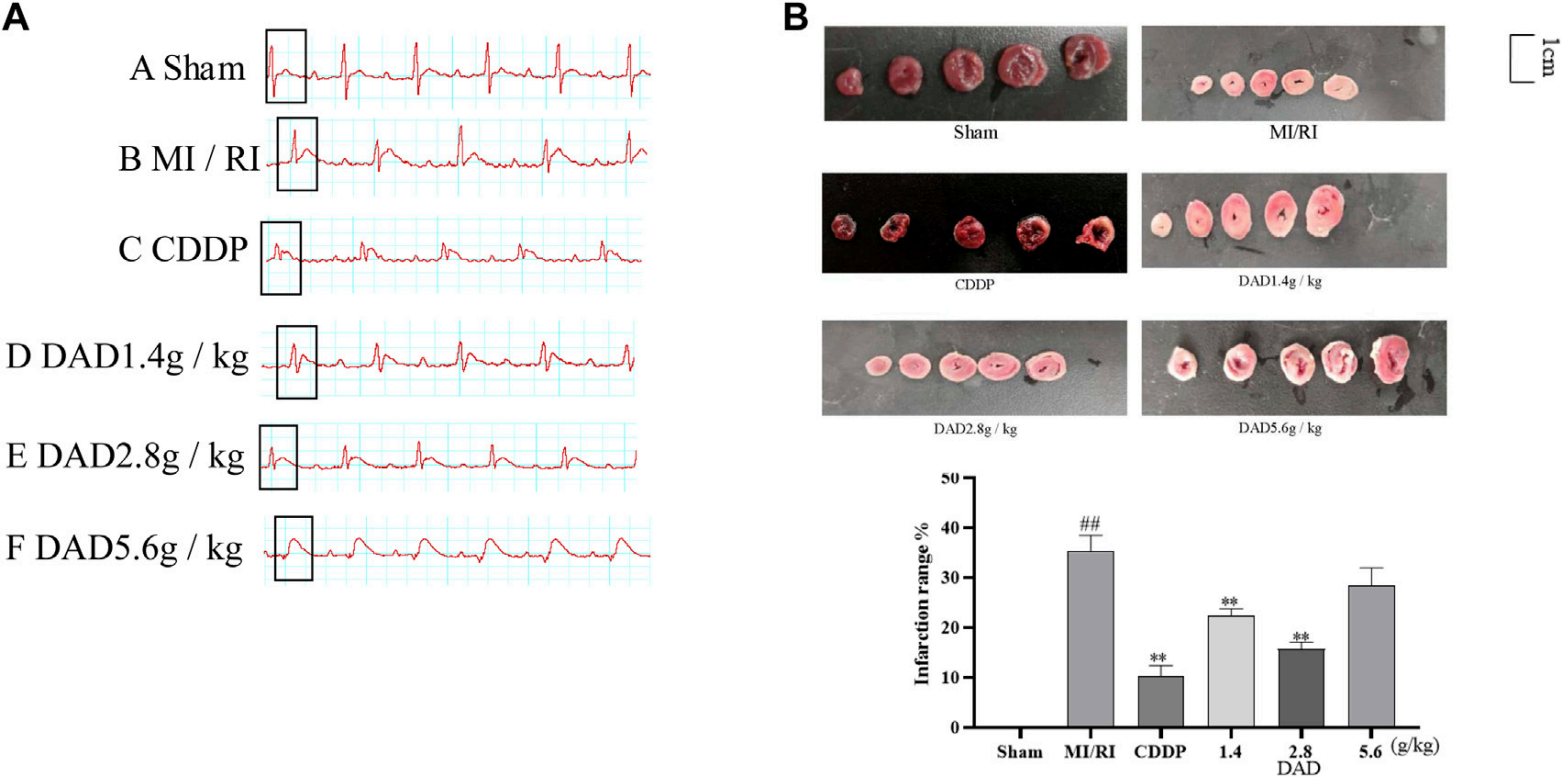
Changes of electrocardiogram in MI/RI rats and effects dried ginger-aconite decoction (DAD) on myocardial infarction area in MI/RI rats. **(A)** After modeling, ECG changes in Sham group, MI/RI group, CDDP group and DAD (1.4 g/kg, 2.8 g/kg, 5.6 g/kg) groups. **(B)** Effects on infarction range reduction of I/R injured rats. Data were presented as mean standard deviation (SD). ^#^ *p* < 0.05, ^##^ *p* < 0.01 vs. sham group. *p < 0.05, **p < 0.01 vs. MI/RI group.

The electrocardiogram test results (Figure 5A) of rats presented that the ST segment was elevated after reperfusion for each group compared to the sham operation group, indicating that the model had been successfully established. Compared to the sham group, the MI/RI group had significantly increased the infarct size (45%) of the myocardial tissue (Figure 5B), (*p* < 0.01). Compared to the MI/RI group, the infarct size of the myocardial tissue for each administration group had significantly reduced. The lowest infarct size was 9.2% in the CDDP (positive) group. Among the three DAD groups (*p* < 0.01), the MI area of rats in the DAD (2.8 g/kg) medium dose group was the lowest (19.3%) (*p* < 0.01). Meanwhile, although the high dose of DAD (5.6 g/kg) did not manifest a significant reduction in infarct size, a protective trend of infarct size reduction could be perceived.

#### Histopathological Examinations

**FIGURE 6:**
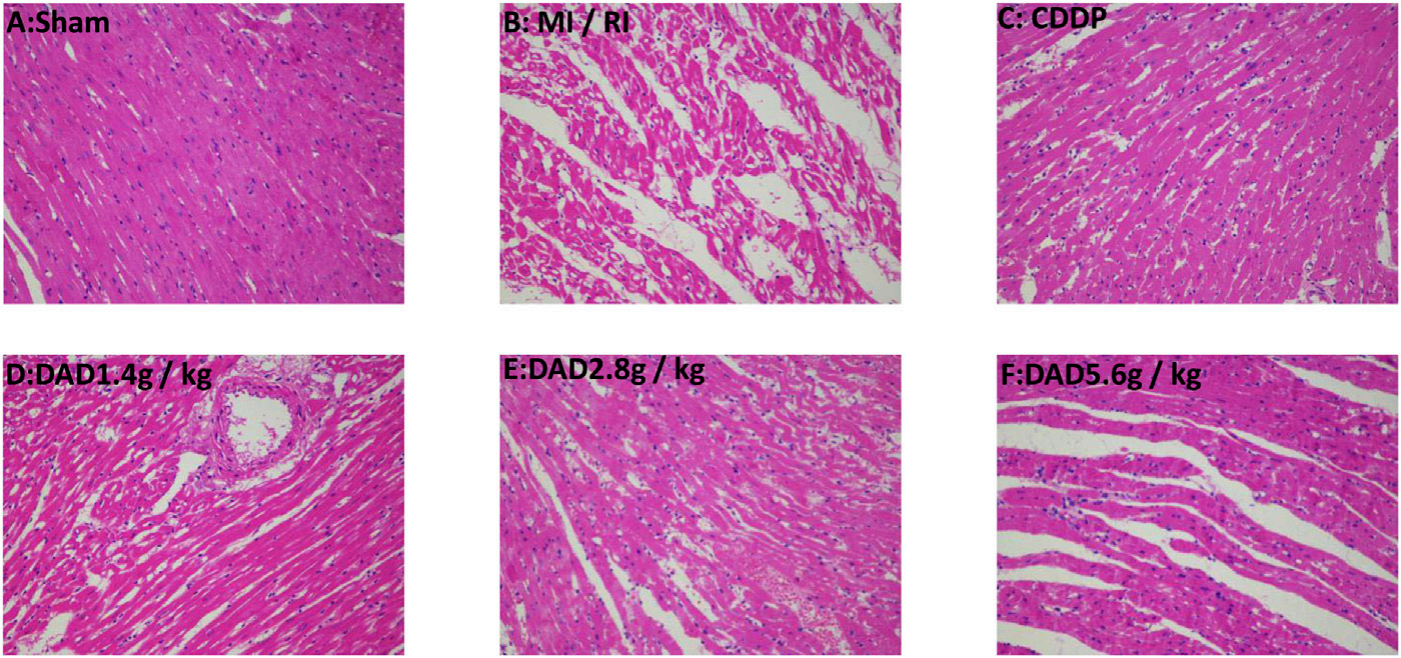
Representative myocardial tissue histopathological sections on the effects of dried ginger-aconite decoction on myocardial infarction size in MI/RI rats (200×). Myocardial tissue injury was assessed by hematoxylin-eosin (H&E) staining.

The degree of myocardial injury could be determined by histopathological examinations (Zhou et al., 2020). In the sham group, the myocardial tissue was intact, with a clear texture and regular arrangement of the myocardial fibers, and without apparent cell swelling and fracture; the nuclei material was evenly distributed, without apparent pathological changes. In contrast, in the MI/RI group, the texture of the myocardial tissue was blurred, the shape of the myocardial fiber was disordered, the myocardial tissue was faulted, the interstitium was severely swollen, the nuclear morphology was changed, and some of the nuclei had disappeared. DAD treatment (1.4, 2.8 and 1.4 g/kg groups) partially attenuated the myocardial tissue histopathological damages, with the greatest improvement realized in the 2.8 g/kg group (Figure 6).

#### Effect of DAD on Myocardial Cell Apoptosis in MI/RI Rats

**FIGURE 7:**
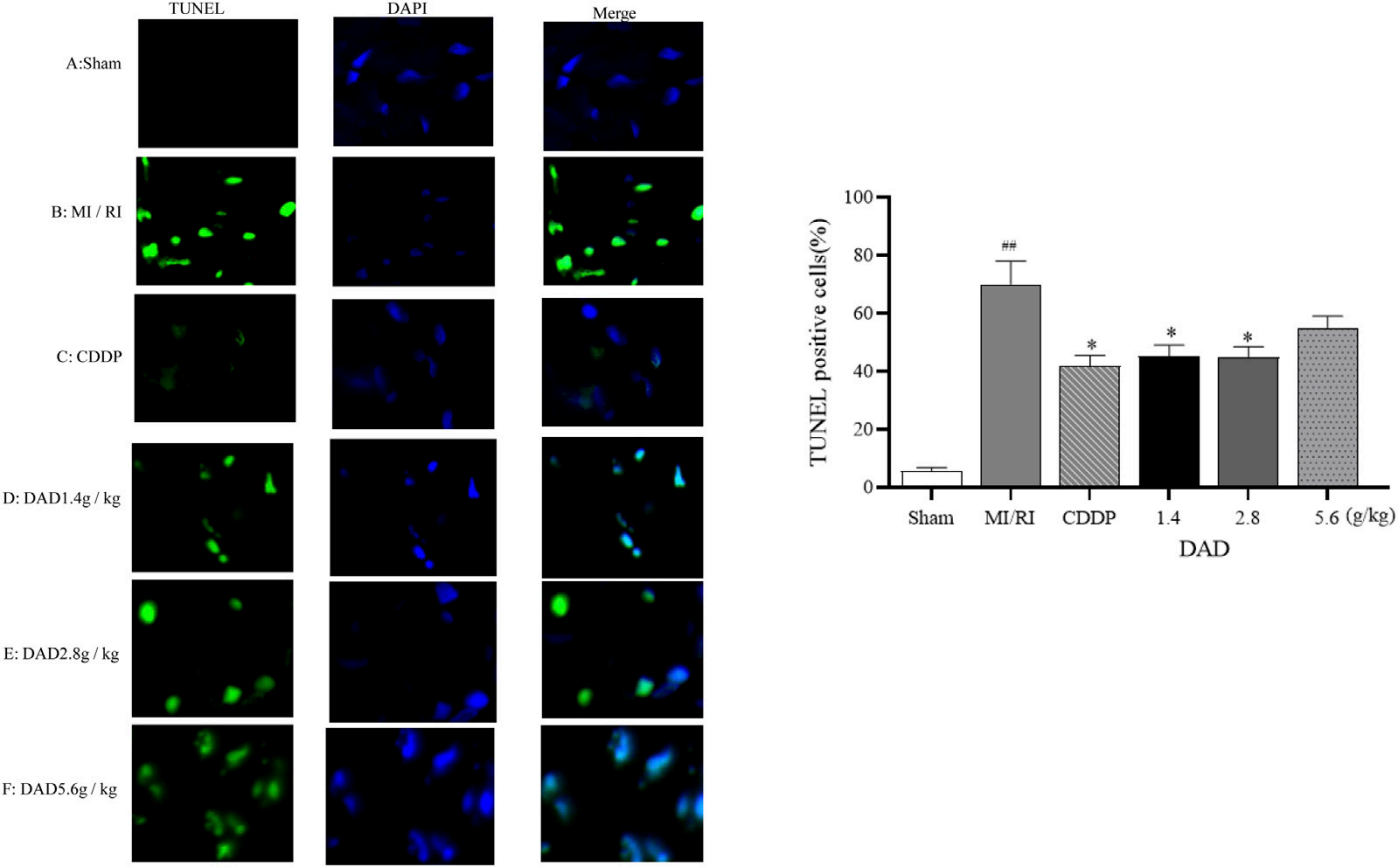
Effects of dried ginger-aconite decoction (DAD) on apoptosis of myocardial cells in MI/RI rats. Apoptosis of cardiomyocytes (green) and DAPI (nuclei Blue). Representative images were shown, and the scale bar indicated 20 µm. The percent of apoptosis cells were calculated for statistical analysis. Data were presented as mean standard deviation (SD). ^#^ *p* < 0.05, ^##^ *p* < 0.01 vs. sham group. *p < 0.05, **p < 0.01 vs. MI/RI group.

TUNEL assay was applied to evaluate the effects of DAD on the apoptosis of myocardial tissue cells in MI/RI rats. Compared to that of the sham operation group, the apoptosis rate (70%) of the MI/RI group had significantly increased (*p* < 0.01). DAD (1.4, 2.8 and 5.6 g/kg) treatment had significantly mitigated the increased percentage of apoptotic cells compared to the model group (*p* < 0.01). Among the three DAD groups, the 2.8 g/kg group exhibited the lowest apoptosis rate (45%). Meanwhile, although a high dose of DAD (5.6 g/kg) did not significantly reduce the apoptosis rate, a decreasing trend in such rate was observed (Figure 7).

#### Results of Biochemical Testing

**FIGURE 8:**
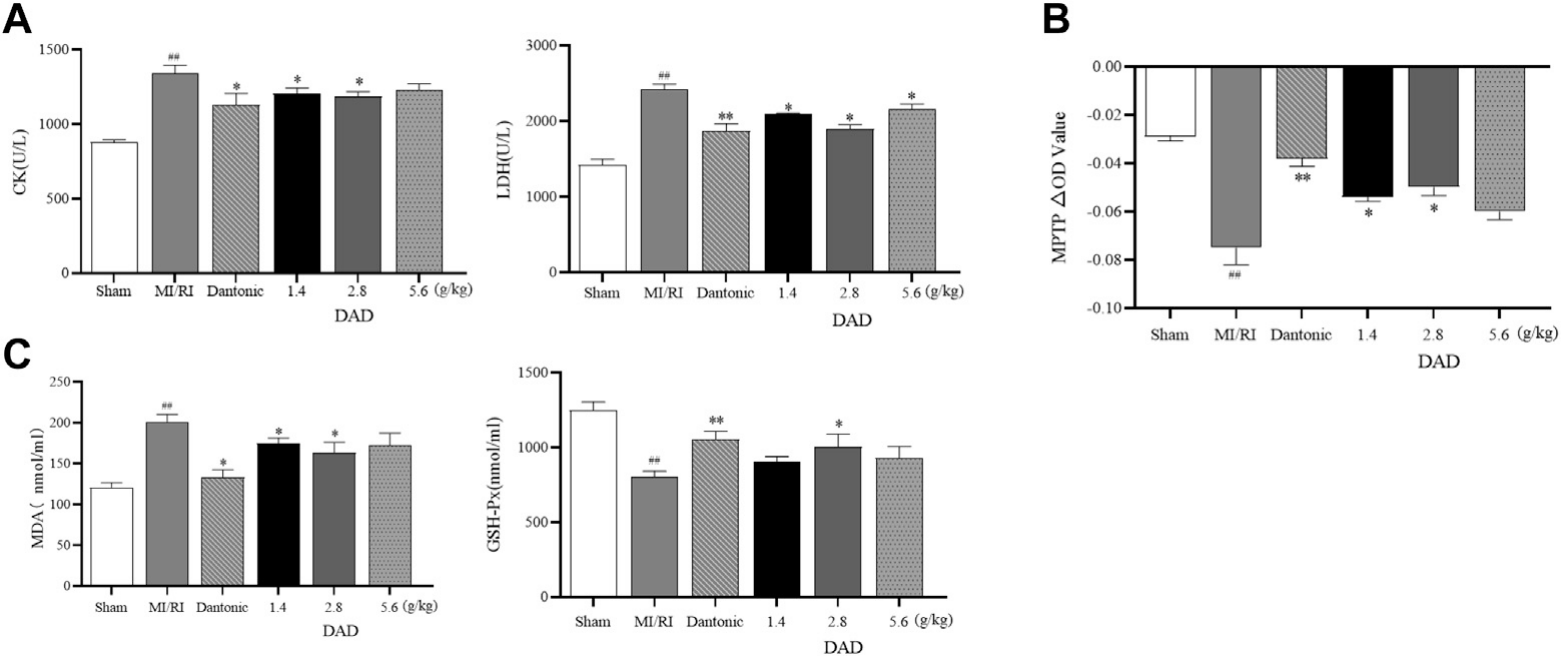
Effects of dried ginger-aconite decoction (DAD) on biochemical indices and MPTP conversion pores in MI/RI rats. **(A)** Effects on activities of myocardial enzymes CK, and LDH. **(B)** Effects on mitochondrial transformation pore MPTP. **(C)** Effect on oxidative stress factors MDA, and GSH-Px. Data were presented as mean standard deviation (SD). #p < 0.05, ##p < 0.01 vs. sham group. *p < 0.05, **p < 0.01 vs. MI/RI group.

Myocardial enzymes are vital indicators in the clinical detection of heart health (Radhiga et al., 2012; Xiang-Qian et al., 2019). The activity of the LDH and CK enzyme sharply increased after myocardial injury. The expressions of LDH and CK significantly increased in the MI/RI group (*p* < 0.01), suggesting that serious heart damage might occur. After treatment, the activities of CK and LDH decreased in each dose group of DADs (1.4, 2.8 and 5.6 g/kg), while the activities of the 2.8 g/kg group had significantly decreased (*p* < 0.01) (Figure 8A). Oxidative stress injury is a key mechanism of I/R injury. Under ischemia and hypoxia conditions, the mitochondria of cardiomyocytes are damaged, the permeability of the mitochondria membrane is transformed (Figure 8B), and reactive oxygen species are released into the cytoplasm through the damaged mitochondria. SOD and GSH-Px are known as free-radical scavengers *in vivo*. Remarkably, after MI/RI, mitochondrial swelling degree and MDA had increased alongside a decreased GSH-Px activity. After treatment with varied DAD (1.4, 2.8 and 5.6 g/kg), the degree of mitochondrial swelling and the degree of elevated MDA level among MI/RI rats were reduced, while the activity of GSH-Px, SOD were restored (Figure 8C). Among the three DAD groups, DAD (2.8 g/kg) manifested the best therapeutic effect (*p* < 0.05).

#### Promotion of PI3K/AKT/GSK-3β by DAD

**FIGURE 9:**
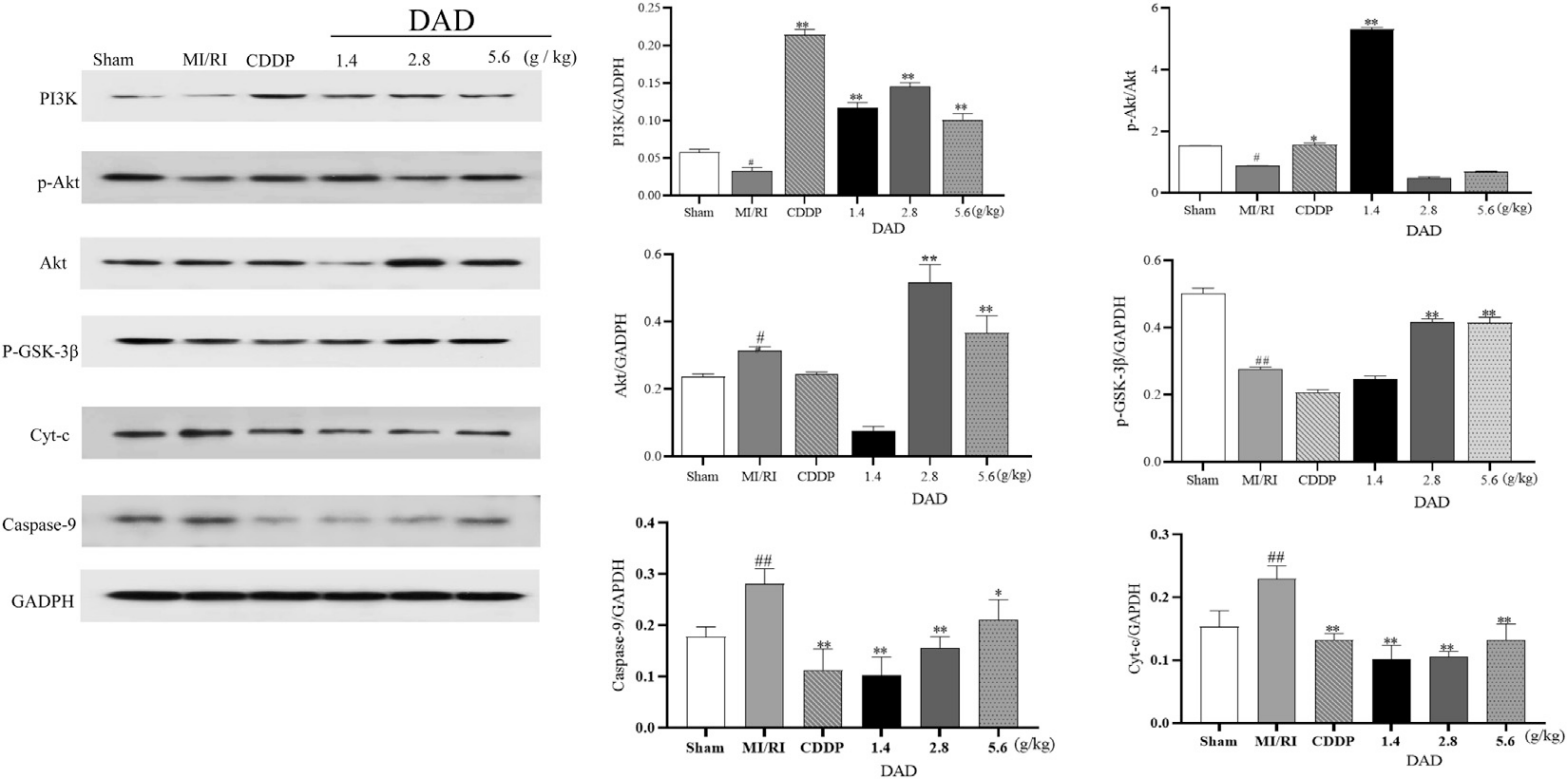
Experimental validation of key signaling pathways and mitochondrial key targets *in vivo*. Dried ginger-aconite decoction(DAD) activates the PI3k/Akt/GSK-3β signaling pathway and inhibits the expression of Cyt-C and CASP9. Data were presented as mean standard deviation (SD) of three independent experiments. ^#^ *p* < 0.05, ^##^ *p* < 0.01 vs. sham group. *p < 0.05, **p < 0.01 vs. MI/RI group.

As a unique molecular target in the mitochondria, Cyt-C can activate apoptosis factors such as CASP9 and can lead to the apoptosis of damaged myocardium (Gao et al., 2016). Considering that the anti-MI/RI mechanism of DAD has been shown to be associated with apoptosis *in vitro*, the network pharmacologically predicted pathway and the related mitochondrial targets *in vivo* were further validated, namely the PI3K/AKT/GSK-3β pathway and the mitochondrial targets–Cyt-C and CASP9. Western blot analysis presented that the expression of PI3K/AKT/GSK-3β was inhibited (*p* < 0.05), and that the expression of Cyt-C and CASP9 was significantly increased in the MI/RI group compared to the sham group (*p* < 0.05). After DAD intervention, the expression of PI3K/AKT/GSK-3β was significantly activated, and the expressions of Cyt-C and CASP9 were significantly decreased in the DAD groups compared to the MI/RI group (Figure 9). Among the three DAD groups, DAD (2.8 g/kg) manifested the best therapeutic effect (*p* < 0.05).

## Discussion

MI/RI is not only a primary cause of death among patients with cardiovascular and cerebrovascular diseases, but can also seriously affect the prognosis of patients with ischemic heart diseases. While the effects of DAD against MI/RI have been demonstrated, its exact mechanism is vague. In this case, pharmacological approaches are adopted to explore relevant molecular pharmacological mechanisms and validate them empirically.

Sixteen active components and 171 targets of DAD were obtained using OB and DL parameters as significant evaluation indices and supplements of significant components. A higher degree of the compound in the C-T network denotes a greater significance. This study found that the degree values of aconitine, 6-ginger, hypaconitine and mesaconitine were among the top. These could be the key components of DAD against MI/RI. The HPLC method was used to determine the above components in DAD that were predicted by network pharmacology. We found that DAD contained aconitine, 6-ginger, mesaconitine, which was consistent with the results of network pharmacology.

Through network pharmacology, it was found that BP that was highly correlated with DAD anti-MI/RI was the negative regulation of the apoptotic process, the lipopolysaccharide-mediated signaling pathways, the regulation of mitochondrial membrane potential, the inherent apoptotic signaling pathway as a response to DNA damage, the external apoptotic signaling pathway lacking ligand, and release of cytochrome C from mitochondria. An analysis of the C-T-P network revealed that DAD anti-MI/RI acted on multiple targets and signaling pathways. The core targets of the active compounds in DAD were determined, namely AKT1, PIK3G, MAPK3, MAPK1, NFKB, TNF, NFKBA, MTOR, GSK3β and TP53. As with BP, these targets were associated with apoptosis and inflammation. Various studies have likewise confirmed that apoptosis is the key mechanism of anti-MI/RI (Zhang et al., 2018b; Li et al., 2020b). Apoptosis plays a vital function in MI/RI prognosis. Studies have determined that the inhibition of myocardial cell apoptosis during MI/RI can mitigate the enlargement of the infarct area and can effectively protect cardiac functions (Geng et al., 2020). Significantly, PI3K/AKT/GSK-3β, an apoptotically-related signaling pathway, has the highest anti-MI/RI correlation in DAD (Chen et al., 2017). Therefore, DAD may play an anti-MI/RI function by inhibiting myocardial apoptosis through the PI3K/AKT/GSK-3β signaling pathway. To further validate this hypothesis, *in vivo* and *in vitro* experiments are conducted to validate its mechanism on network pharmacological prediction.

Mitochondria is the energy factory of the cells and is also the site of ATP synthesis (Wang et al., 2020). On a physiological level, a stable mitochondria provides ATP to the body; when it is damaged (i.e., by hypoxia injury), it can produce superoxides and reactive oxygen species, leading to adverse stimuli like calcium overload and oxidative stress, and further inducing apoptosis and necrosis in cells (Latini et al., 2015). The abnormal openness of MPTP, as a key regulator of mitochondrial functions, can induce mitochondrial structure disorders, which influence mitochondrial functions and eventually result to cell apoptosis. Under normal physiological conditions, MPTP remains closed, while Ca^2+^ overload and excessive oxidative stress can induce it to open (Tait and Green, 2010). Cyt-C generally exists in the space between the inner and outer membranes of the mitochondria, and cannot cross the outer membrane to reach the cytoplasm under physiological conditions (Joseph and Levine, 2015). When the MPTP is abnormally open and causes damage to the mitochondrial membrane structure, Cyt-C is released from the mitochondria into the cytoplasm and acts as a vital pro-apoptotic factor. It binds to the apoptotic protease activator 1 in the synergistic role of deoxyadenosine triphosphate. Caspase-9 is activated, eventually leading to apoptosis (Mace et al., 2014). Interestingly, *in vitro* studies have depicted that DAD reduces apoptosis and increases ATPase activity in H/R-damaged H9C2 cells. Meanwhile, *in vivo* studies have presented that DAD can reduce myocardial injury in MI/RI rats, with the rate of apoptosis of myocardial cells, the oxidative damage, the degree of mitochondrial MPTP opening, and the expressions of Cyt-C and CASP9 likewise all reduced. Therefore, as predicted by network pharmacological analysis, it was verified that DAD could reduce cardiomyocyte apoptosis both *in vivo* and *in vitro*.

The PI3K/AKT/GSK-3β signaling pathway is a fundamental pathway in MI/RI. Phosphatidylinositol 3-kinase (PI3K, a lipid kinase) can specifically catalyze the phosphorylation of the phosphatidylinositol-3 hydroxyl group (Stokes and Condliffe, 2018). It phosphorylates PIP2 to produce PIP3 firstly (Zhang et al., 2017), and then activates AKT (Chen et al., 2014). Activated AKT can yield a series of phosphorylation cascade reactions and can regulate significant downstream effector molecules such as Glycogen synthase kinase-3β (GSK-3β) to exert their biological functions (Ya-Fei et al., 2010). GSK-3β is a serine/threonine kinase (Sun et al., 2011; Barré and Perkins, 2014), and is the most extensively studied downstream target of AKT. It can promote cardiomyocyte apoptosis through an intrinsic mitochondrial pathway (Yan et al., 2011); meanwhile, phosphorylated GSK3β has no biological activity, which can reduce myocardial cell apoptosis. (Jun et al., 2011). The PI3K/AKT/GSK-3β signaling pathway plays a vital function in the growth, survival, apoptosis and proliferation of cells. Recent studies have presented that the activation of this signaling pathway can reduce body damage caused by hypoxia (Kaneko et al., 2016; Li et al., 2018; Jing et al., 2019). Interestingly, *in vivo* studies have indicated that DAD can activate the expression of the said signaling pathway.

The innovation of this study involves the prediction of active components, BP and mechanism of action of DAD against MI/RI using network pharmacology. This study has demonstrated that DAD plays an anti-MI/RI role by activating PI3K/AKT/GSK3β to reduce cardiomyocyte apoptosis. Nevertheless, the limitations of this study should be acknowledged. Firstly, DAD at its highest concentration has either no or minimal effect against MI/RI. In the dose-setting process, the clinical equivalent dose was selected as the medium-dose group. In Figures 3, 5–9, dose dependence was not found, which might be because the concentration gradient established was not large enough. Future studies may focus on the study of the “dose-effect” relationship of DAD in regulating SOD and MDA, as well as other indices. Moreover, most TCMs can play multiple therapeutic roles, and network pharmacology can predict DAD anti-MI/RI by inflammation relevant signaling pathways. Thus, further studies may explore inflammation-related signaling pathways and regulators. In addition, the active compounds neutralized in DAD have been identified by network pharmacology. However, the compounds that exert therapeutic effects are still unknown and deserve further study. Overall, the aforementioned limitations should continue to be studied in order to clarify the therapeutic mechanisms of DAD.

## Conclusion

In this study, a comprehensive strategy that involved network pharmacological analysis, HPLC technology and experimental verification was adopted to determine the potential active components and molecular mechanisms of DAD against MI/RI. Based on the TCMSP database and on core compounds, 16 active compounds of DAD were obtained. The presence of four of these components was identified in DAD by HPLC, in which the components were potential therapeutic ingredients as predicted by network pharmacology. Through the analysis of BP, hub targets and hub signaling pathways and experimental verification, it was concluded that DAD could play an anti-MI/RI role by inhibiting myocardial apoptosis via PI3K/AKT/GSK3β. The experimental results were consistent with the network pharmacological predictions. Relatively, this study evidently clarified the anti-MI/RI mechanism of DAD, which could provide a certain basis for future studies on DAD.

## Data Availability

The original contributions presented in the study are included in the article/Supplementary Material, further inquiries can be directed to the corresponding author.
