# Supplementary material for: Anti-Myocardial Ischemia Reperfusion Injury Mechanism of Dried Ginger-Aconite Decoction Based on Network Pharmacology
Source: Front Pharmacol. 2021 May 6;12:609702. doi: 10.3389/fphar.2021.609702 (PMC8135102; doi:10.3389/fphar.2021.609702)
Supplement: Supplementary file 1 [file DataSheet1.zip › All the FIG.pptx]

## Slide 1
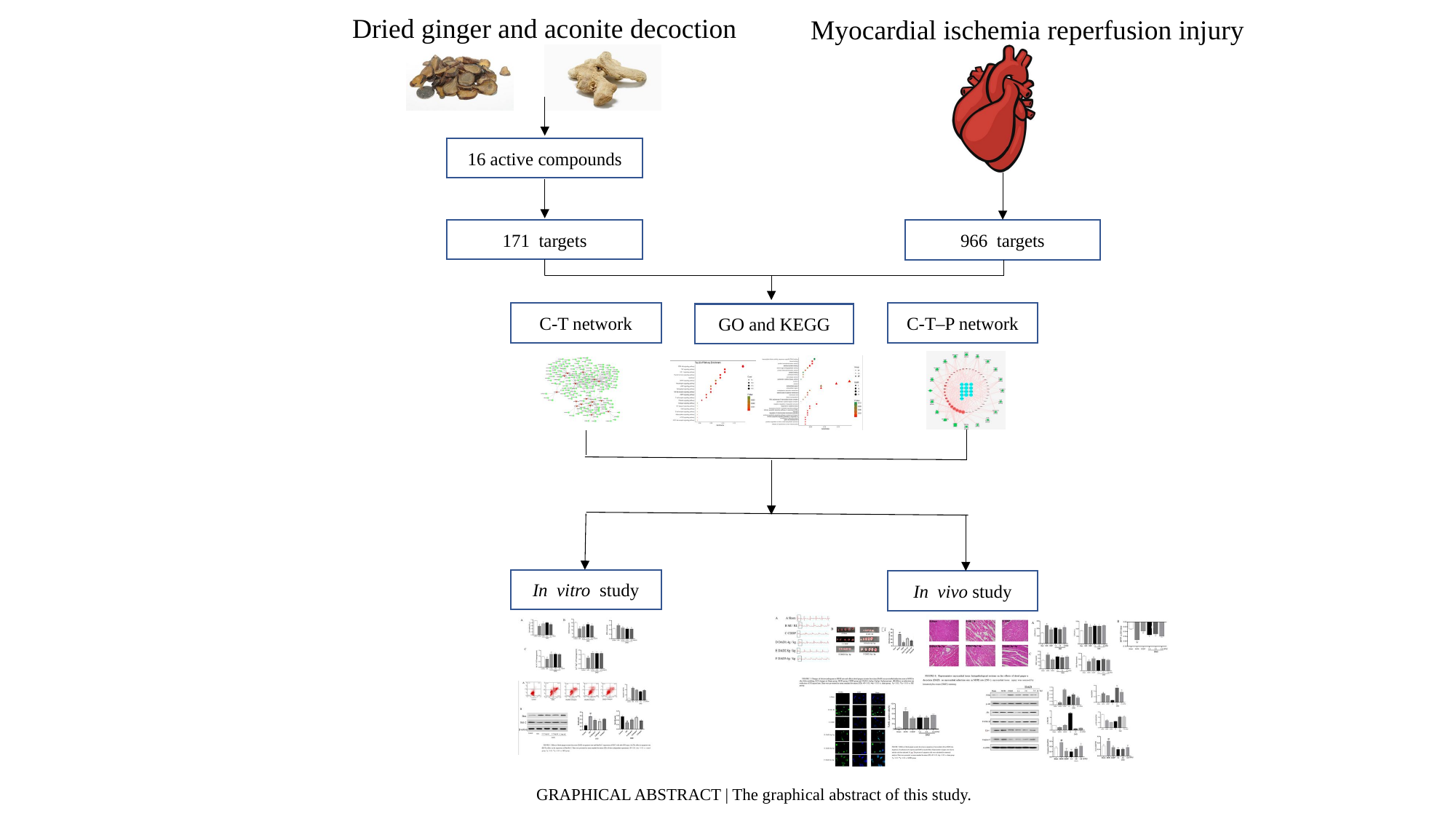

Dried ginger and aconite decoction
Myocardial ischemia reperfusion injury
16 active compounds
171 targets
966 targets
C-T–P network
C-T network
GO and KEGG
In vitro study
In vivo study
GRAPHICAL ABSTRACT | The graphical abstract of this study.

## Slide 2
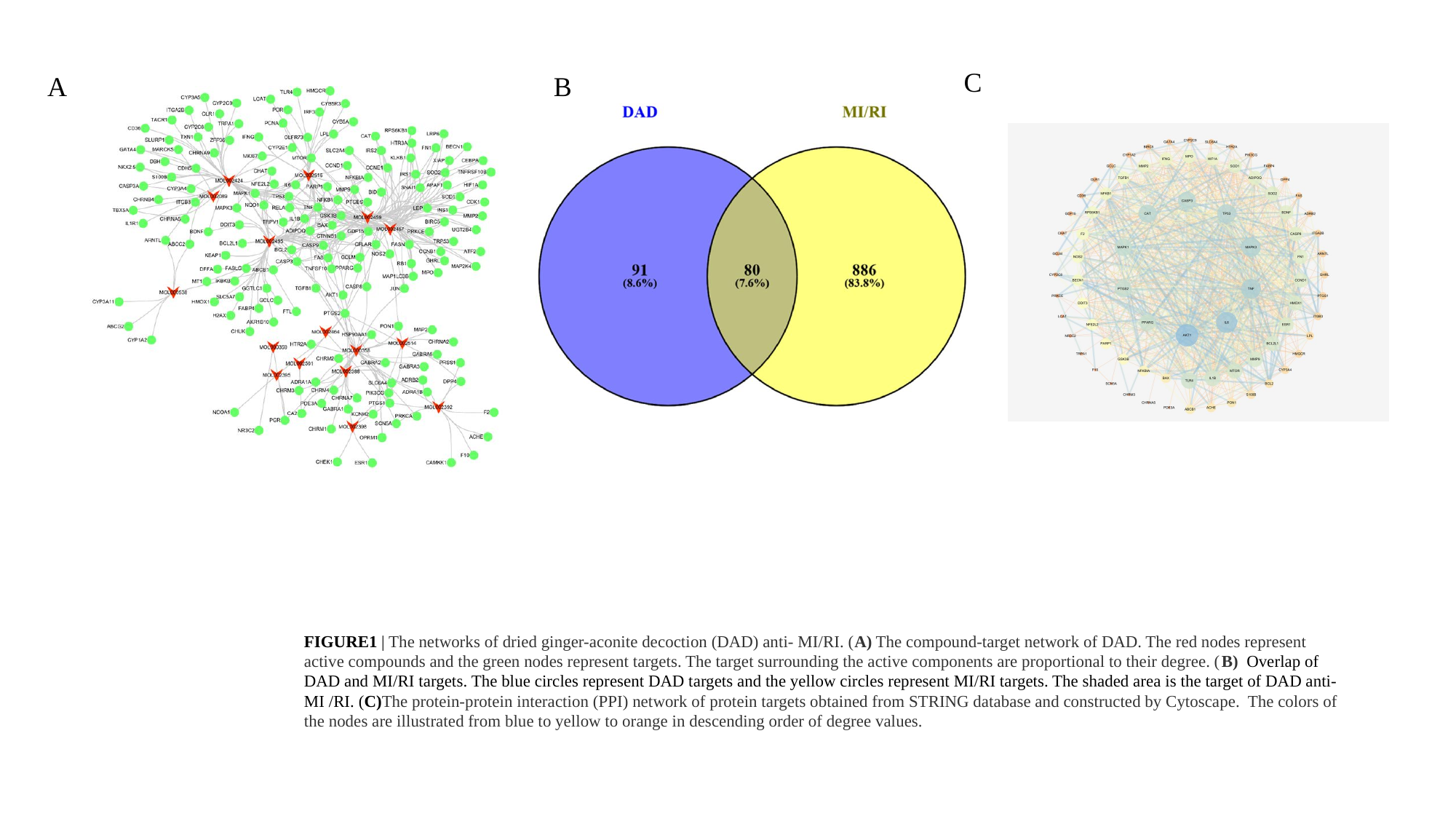

C
A
B
FIGURE1 | The networks of dried ginger-aconite decoction (DAD) anti- MI/RI. (A) The compound-target network of DAD. The red nodes represent active compounds and the green nodes represent targets. The target surrounding the active components are proportional to their degree. (B)  Overlap of DAD and MI/RI targets. The blue circles represent DAD targets and the yellow circles represent MI/RI targets. The shaded area is the target of DAD anti-MI /RI. (C)The protein-protein interaction (PPI) network of protein targets obtained from STRING database and constructed by Cytoscape. The colors of the nodes are illustrated from blue to yellow to orange in descending order of degree values.

## Slide 3
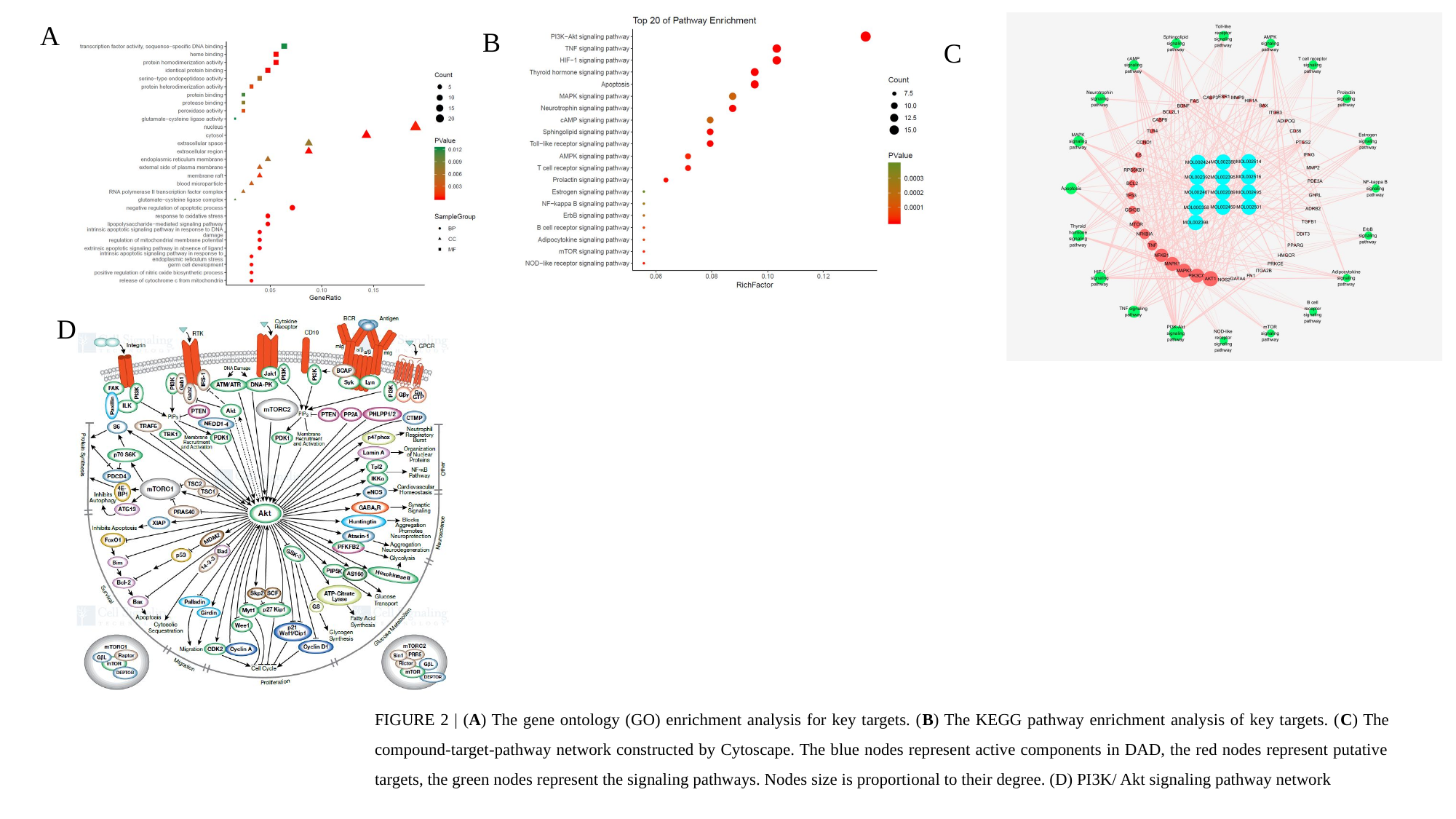

A
B
C
D
FIGURE 2 | (A) The gene ontology (GO) enrichment analysis for key targets. (B) The KEGG pathway enrichment analysis of key targets. (C) The compound-target-pathway network constructed by Cytoscape. The blue nodes represent active components in DAD, the red nodes represent putative targets, the green nodes represent the signaling pathways. Nodes size is proportional to their degree. (D) PI3K/ Akt signaling pathway network

## Slide 4
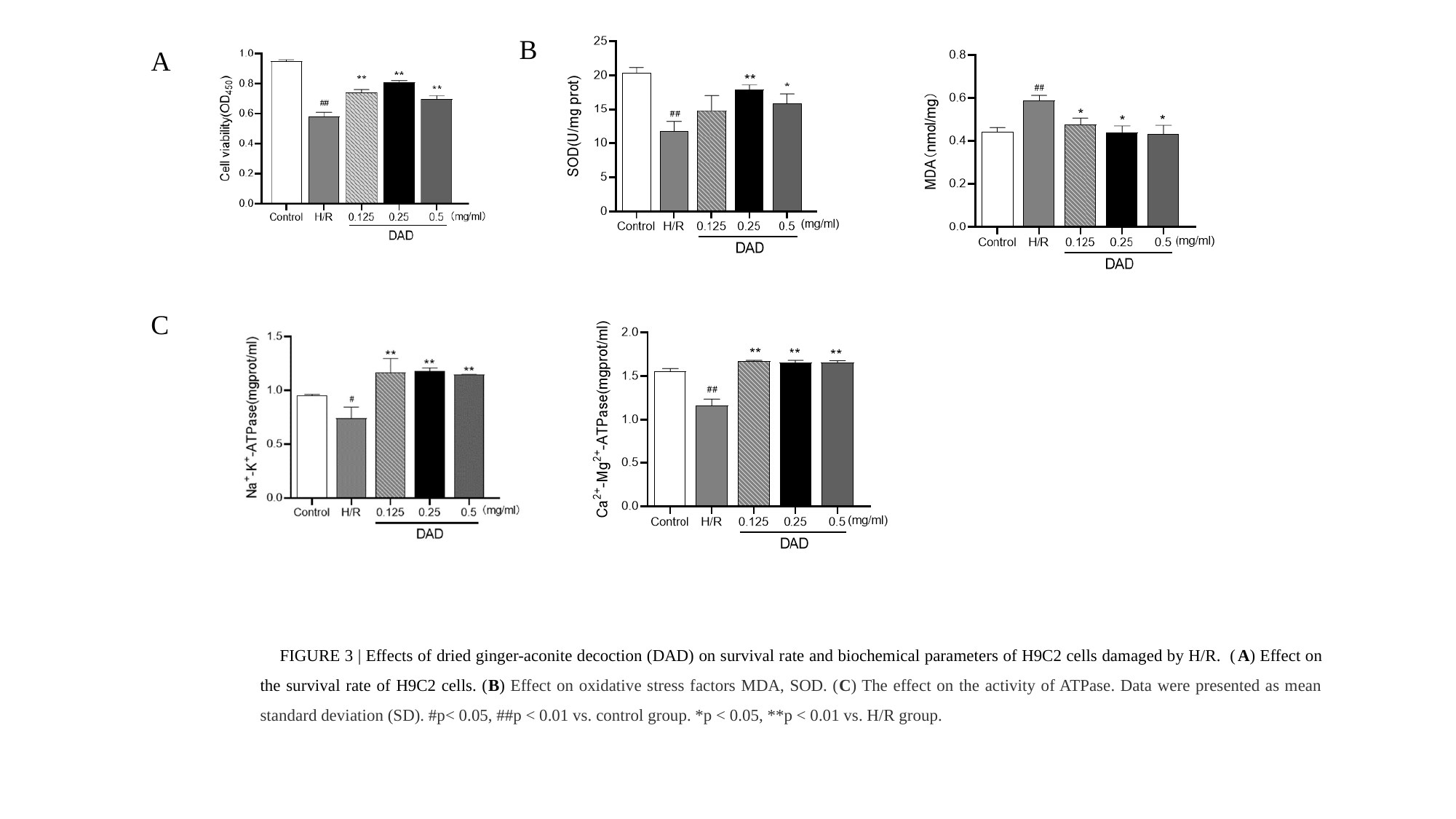

B
A
C
FIGURE 3 | Effects of dried ginger-aconite decoction (DAD) on survival rate and biochemical parameters of H9C2 cells damaged by H/R. (A) Effect on the survival rate of H9C2 cells. (B) Effect on oxidative stress factors MDA, SOD. (C) The effect on the activity of ATPase. Data were presented as mean standard deviation (SD). #p< 0.05, ##p < 0.01 vs. control group. *p < 0.05, **p < 0.01 vs. H/R group.

## Slide 5
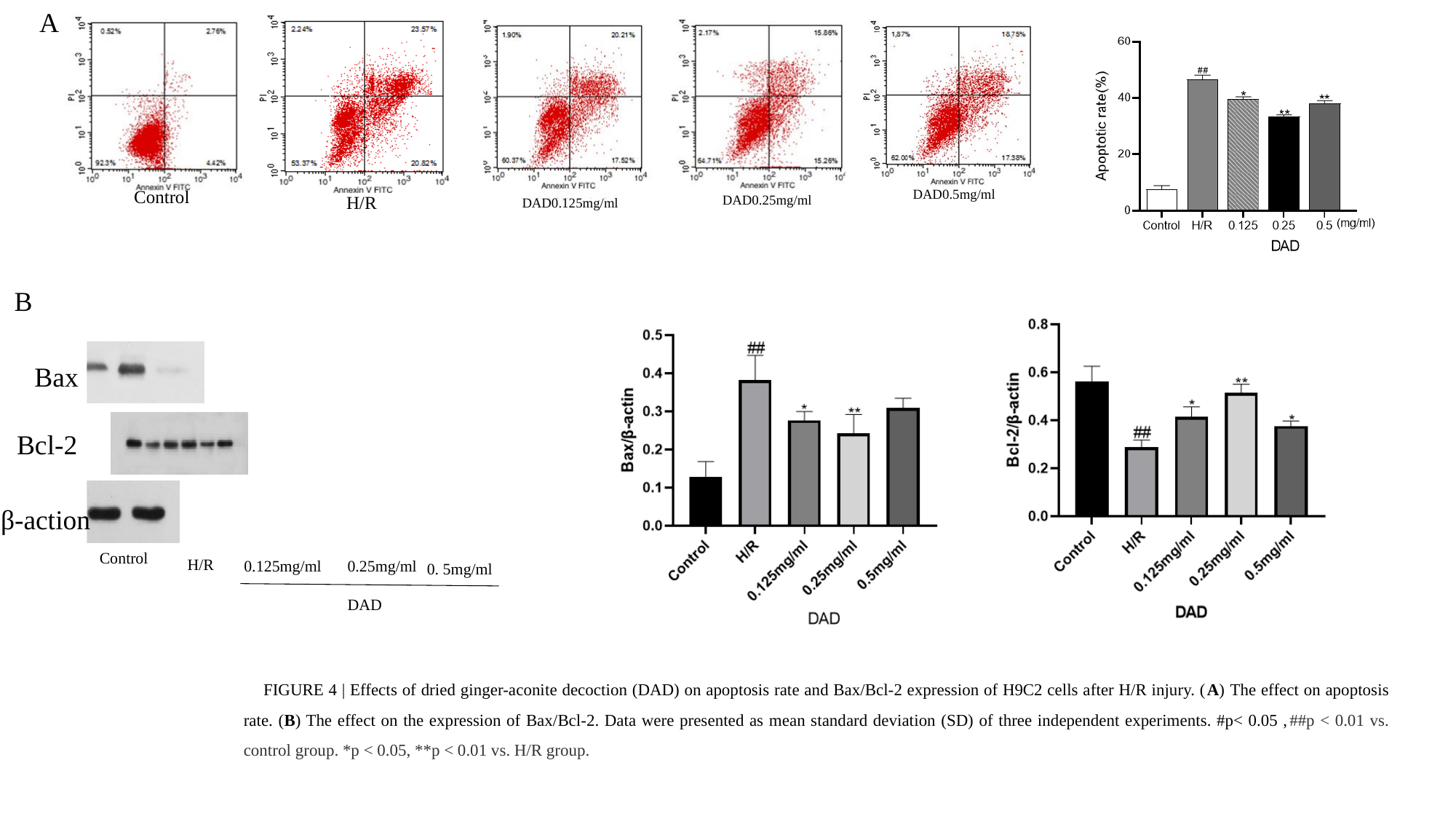

A
Control
DAD0.5mg/ml
H/R
DAD0.25mg/ml
DAD0.125mg/ml
B
Bax
Bcl-2
β-action
Control
H/R
0.125mg/ml
0.25mg/ml
0. 5mg/ml
DAD
 FIGURE 4 | Effects of dried ginger-aconite decoction (DAD) on apoptosis rate and Bax/Bcl-2 expression of H9C2 cells after H/R injury. (A) The effect on apoptosis rate. (B) The effect on the expression of Bax/Bcl-2. Data were presented as mean standard deviation (SD) of three independent experiments. #p< 0.05 ,##p < 0.01 vs. control group. *p < 0.05, **p < 0.01 vs. H/R group.

## Slide 6
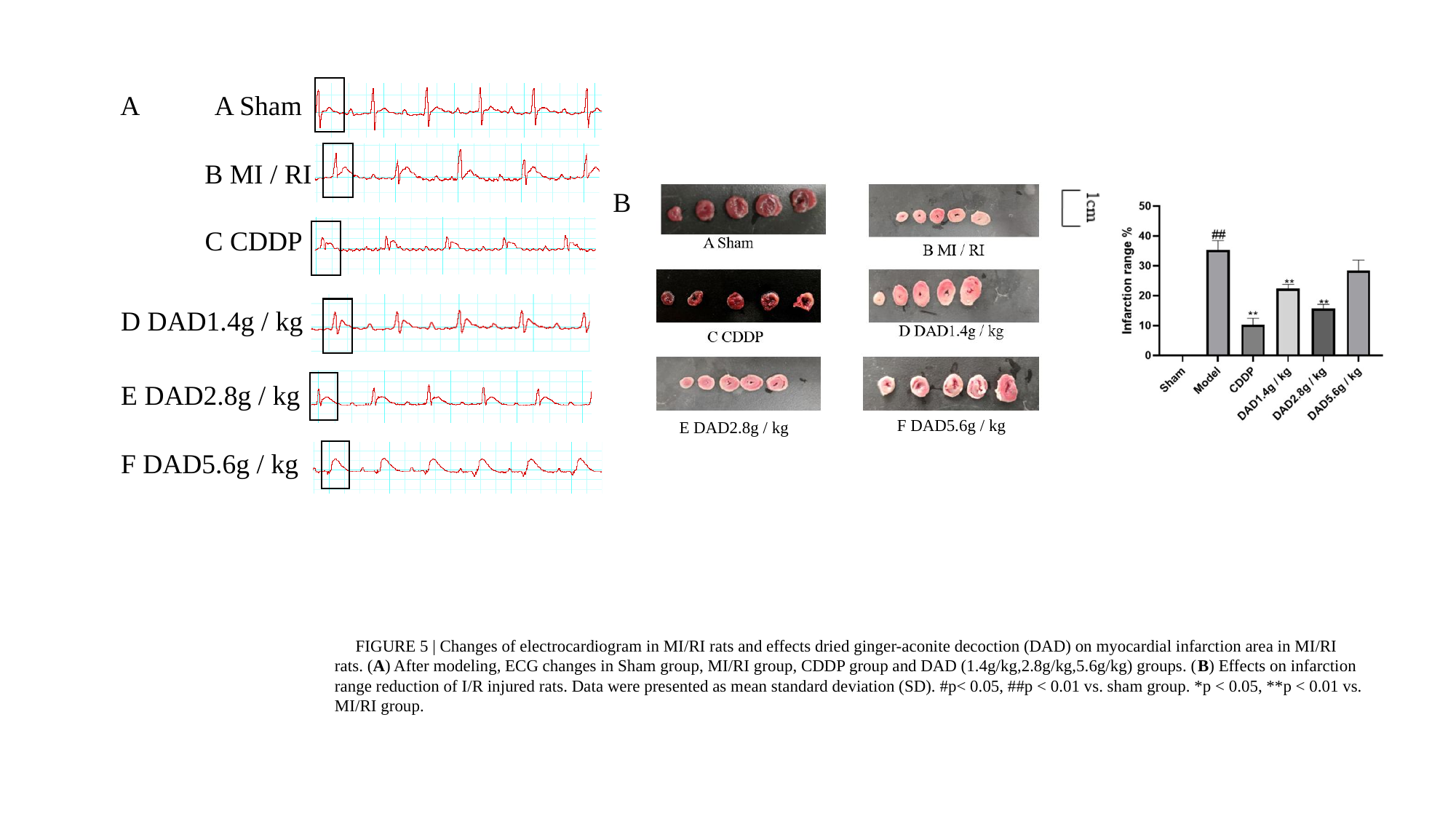

A
A Sham
B MI / RI
B
C CDDP
D DAD1.4g / kg
E DAD2.8g / kg
F DAD5.6g / kg
E DAD2.8g / kg
F DAD5.6g / kg
 FIGURE 5 | Changes of electrocardiogram in MI/RI rats and effects dried ginger-aconite decoction (DAD) on myocardial infarction area in MI/RI rats. (A) After modeling, ECG changes in Sham group, MI/RI group, CDDP group and DAD (1.4g/kg,2.8g/kg,5.6g/kg) groups. (B) Effects on infarction range reduction of I/R injured rats. Data were presented as mean standard deviation (SD). #p< 0.05, ##p < 0.01 vs. sham group. *p < 0.05, **p < 0.01 vs. MI/RI group.

## Slide 7
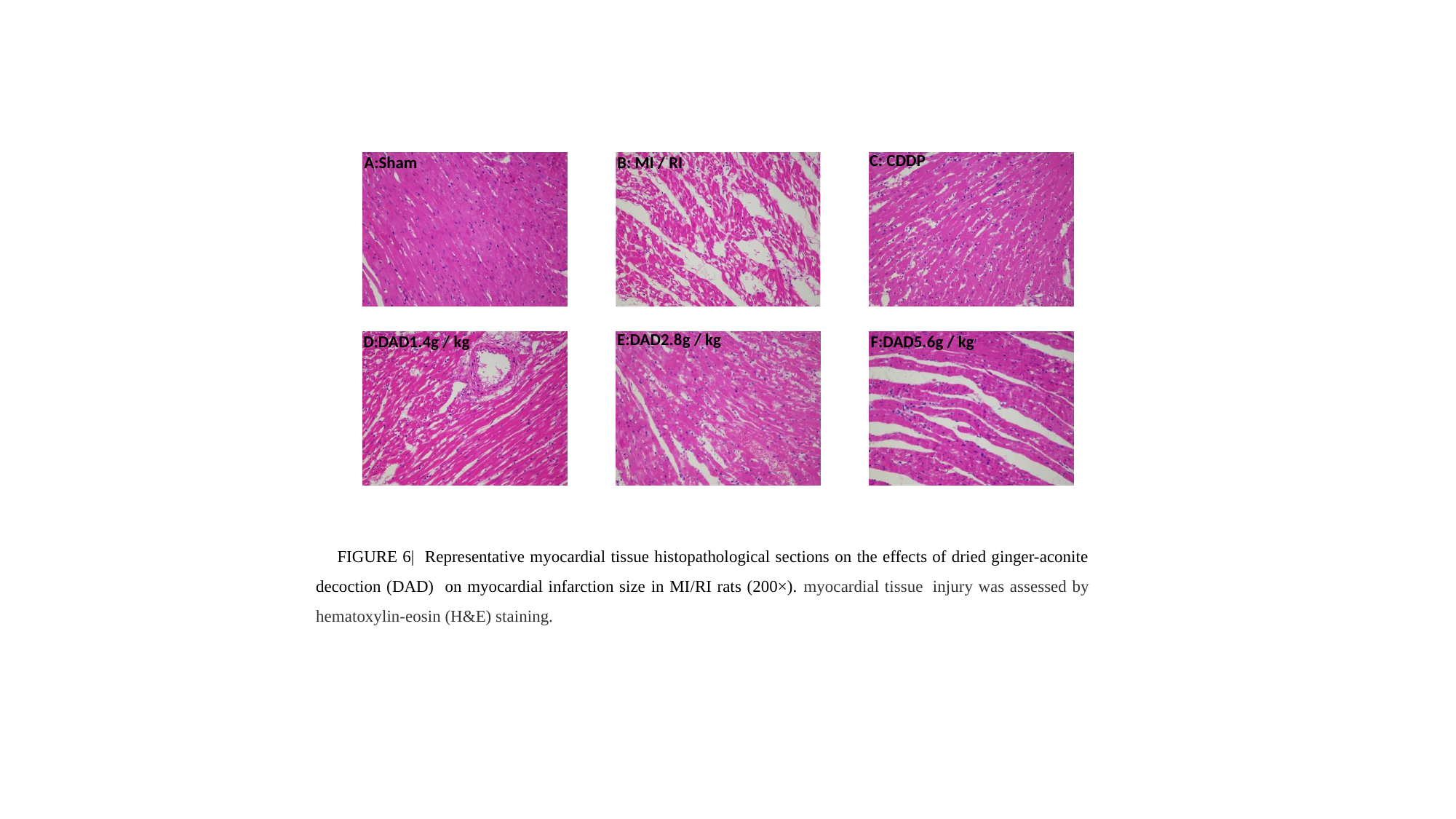

C: CDDP
B: MI / RI
A:Sham
E:DAD2.8g / kg
D:DAD1.4g / kg
F:DAD5.6g / kg
 FIGURE 6| Representative myocardial tissue histopathological sections on the effects of dried ginger-aconite decoction (DAD) on myocardial infarction size in MI/RI rats (200×). myocardial tissue  injury was assessed by hematoxylin-eosin (H&E) staining.

## Slide 8
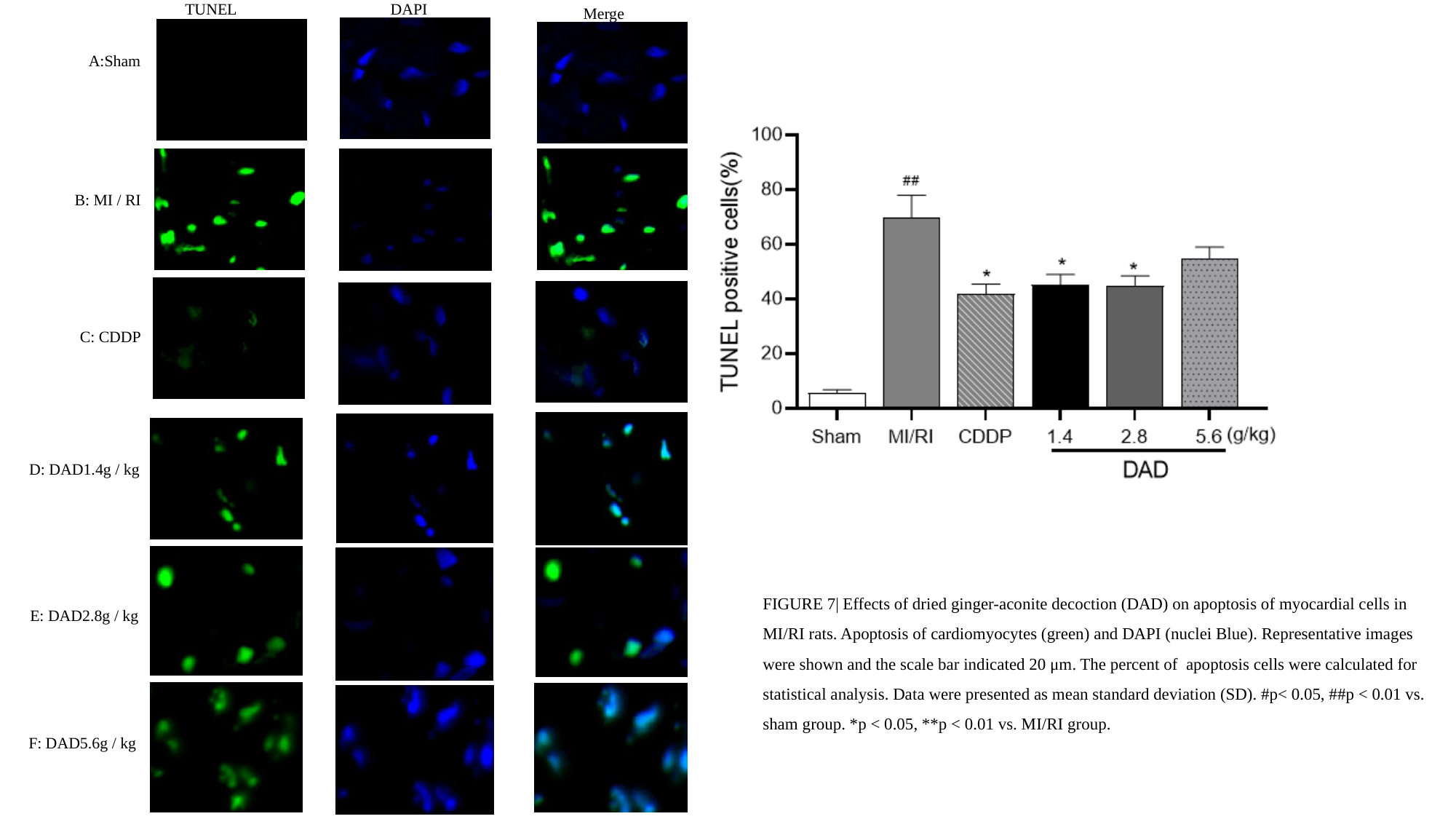

TUNEL
DAPI
Merge
A:Sham
B: MI / RI
C: CDDP
D: DAD1.4g / kg
E: DAD2.8g / kg
FIGURE 7| Effects of dried ginger-aconite decoction (DAD) on apoptosis of myocardial cells in MI/RI rats. Apoptosis of cardiomyocytes (green) and DAPI (nuclei Blue). Representative images were shown and the scale bar indicated 20 μm. The percent of apoptosis cells were calculated for statistical analysis. Data were presented as mean standard deviation (SD). #p< 0.05, ##p < 0.01 vs. sham group. *p < 0.05, **p < 0.01 vs. MI/RI group.
F: DAD5.6g / kg

## Slide 9
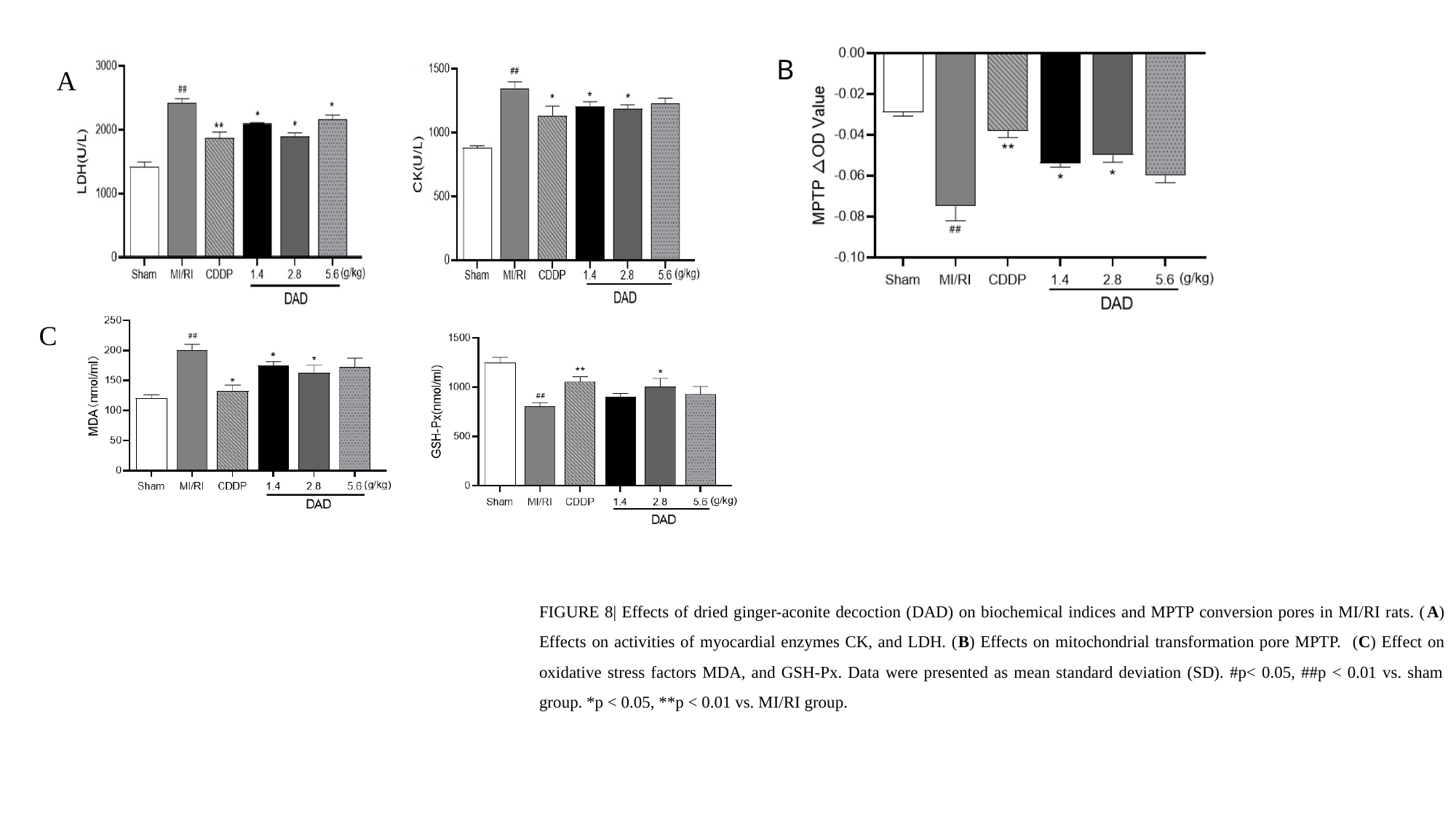

B
A
C
FIGURE 8| Effects of dried ginger-aconite decoction (DAD) on biochemical indices and MPTP conversion pores in MI/RI rats. (A) Effects on activities of myocardial enzymes CK, and LDH. (B) Effects on mitochondrial transformation pore MPTP. (C) Effect on oxidative stress factors MDA, and GSH-Px. Data were presented as mean standard deviation (SD). #p< 0.05, ##p < 0.01 vs. sham group. *p < 0.05, **p < 0.01 vs. MI/RI group.

## Slide 10
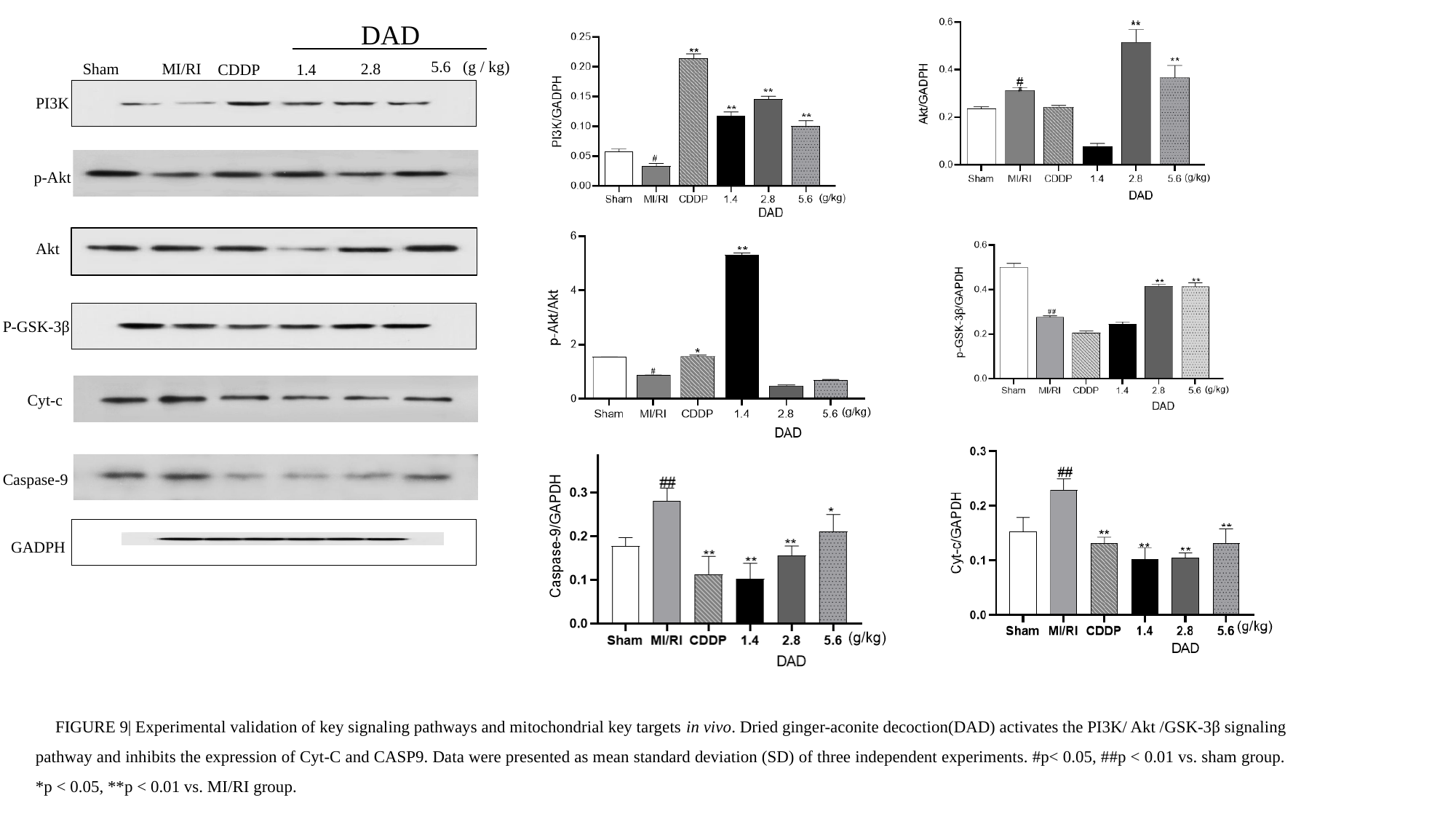

DAD
5.6 (g / kg)
Sham
MI/RI
2.8
CDDP
1.4
PI3K
p-Akt
Akt
P-GSK-3β
Cyt-c
Caspase-9
GADPH
FIGURE 9| Experimental validation of key signaling pathways and mitochondrial key targets in vivo. Dried ginger-aconite decoction(DAD) activates the PI3K/ Akt /GSK-3β signaling pathway and inhibits the expression of Cyt-C and CASP9. Data were presented as mean standard deviation (SD) of three independent experiments. #p< 0.05, ##p < 0.01 vs. sham group. *p < 0.05, **p < 0.01 vs. MI/RI group.

## Slide 11
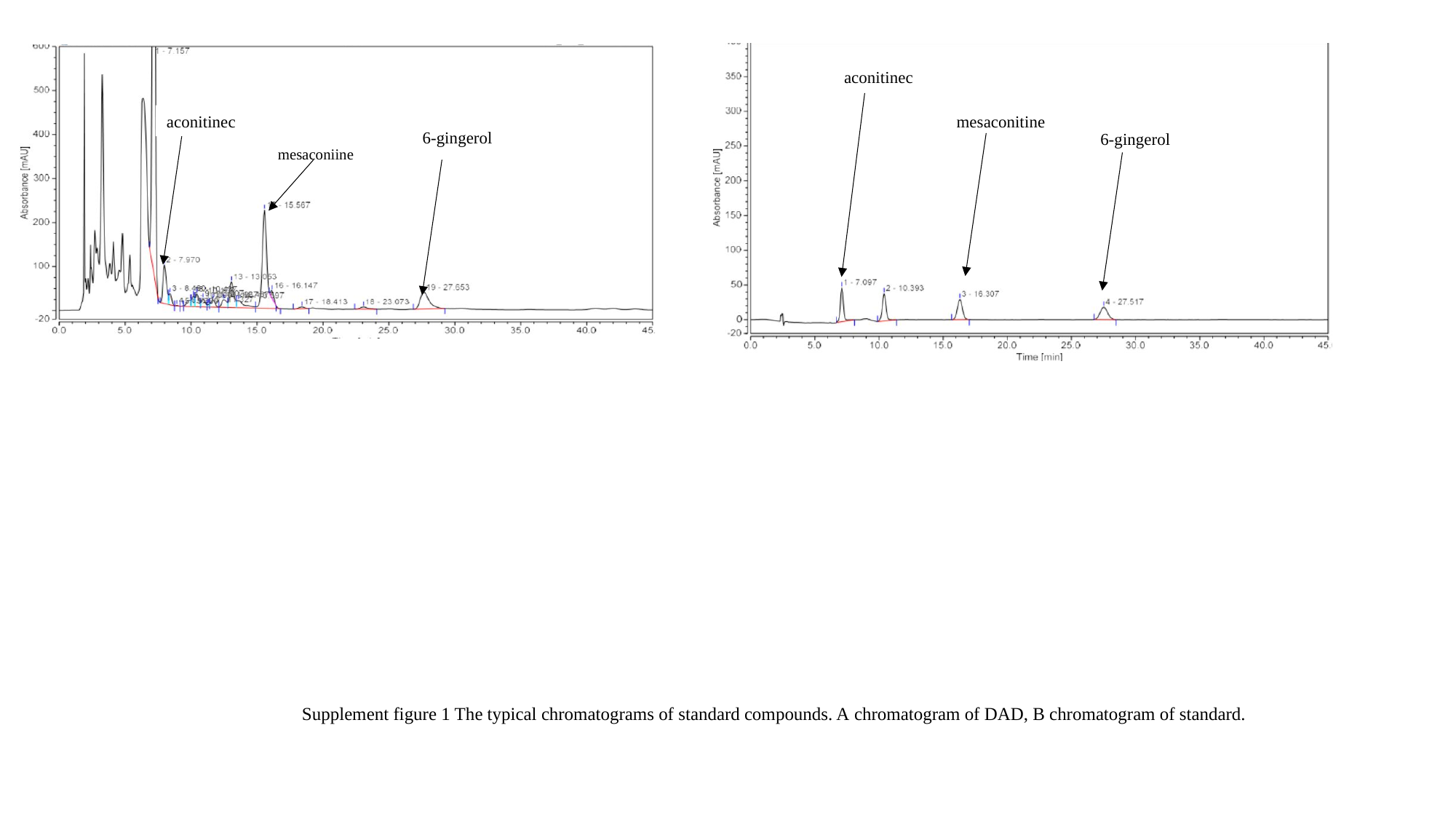

aconitinec
aconitinec
mesaconitine
6-gingerol
6-gingerol
mesaconiine
Supplement figure 1 The typical chromatograms of standard compounds. A chromatogram of DAD, B chromatogram of standard.
