# Supplementary material for: Anti-Myocardial Ischemia Reperfusion Injury Mechanism of Dried Ginger-Aconite Decoction Based on Network Pharmacology
Source: Front Pharmacol. 2021 May 6;12:609702. doi: 10.3389/fphar.2021.609702 (PMC8135102; doi:10.3389/fphar.2021.609702)
Supplement: Supplementary file 2 [file Image1.pdf]

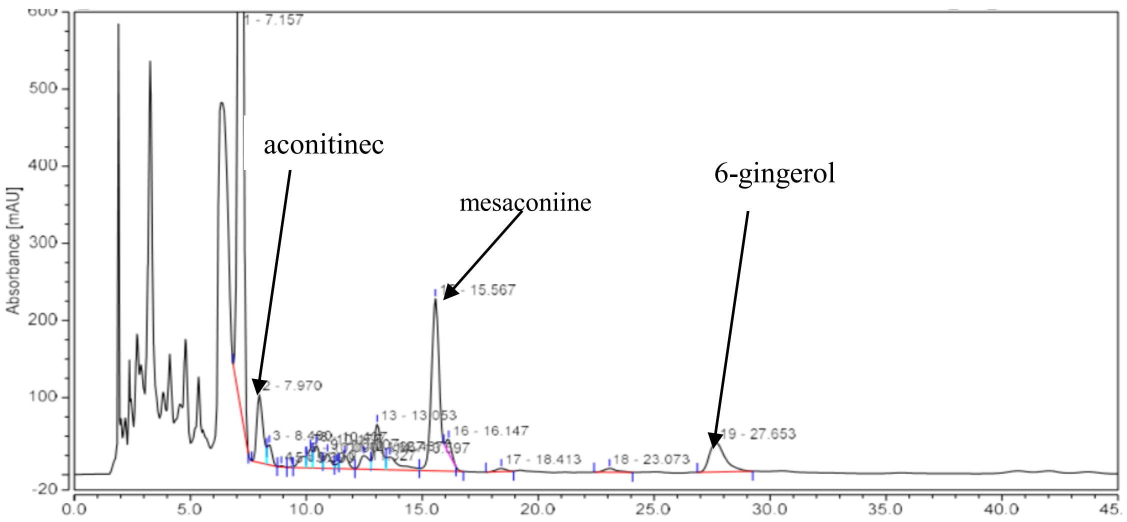

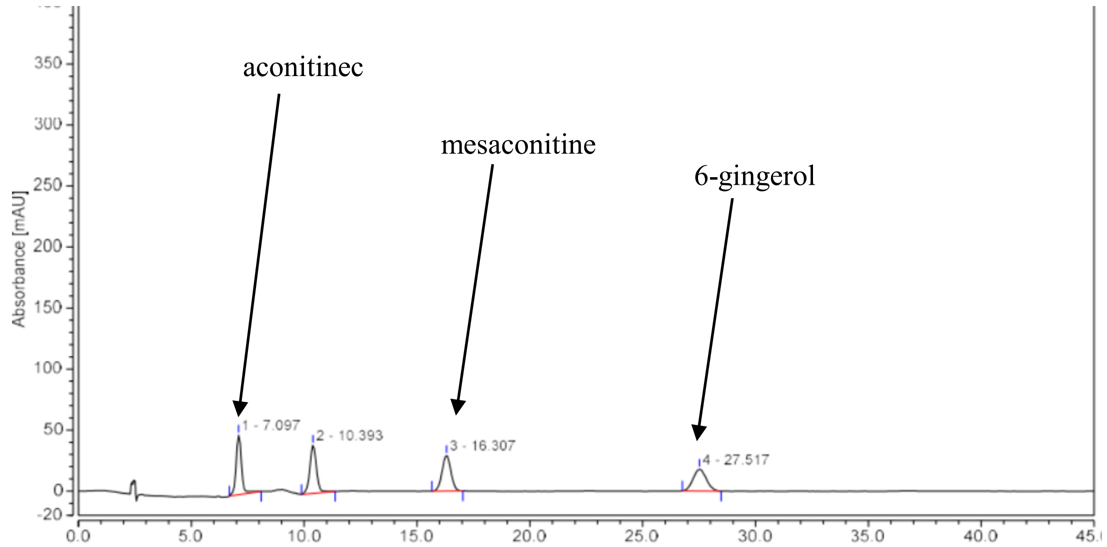

Supplement figure 1 The typical chromatograms of standard compounds. A chromatogram of DAD, B chromatogram of standard.
